# Recent Advances in Synthesis of 4-Arylcoumarins

**DOI:** 10.3390/molecules23102417

**Published:** 2018-09-20

**Authors:** Jong-Wha Jung, Nam-Jung Kim, Hwayoung Yun, Young Taek Han

**Affiliations:** 1 College of Pharmacy, Research Institute of Pharmaceutical Sciences, Kyungpook National University, Daegu 41566, Korea; jungj@knu.ac.kr; 2 College of Pharmacy, Kyung Hee University, Seoul 02453, Korea; kimnj@khu.ac.kr; 3 College of Pharmacy, Pusan National University, Busan 46241, Korea; 4 College of Pharmacy, Dankook University, Cheonan 31116, Korea

**Keywords:** 4-arylcoumarins, Pechmann condensation, hydroarylation, cyclocarbonylation, Wittig-type olefination, aldol-type olefination, cycloisomerization, transition-metal-catalysis

## Abstract

4-Arylcoumarins (4-aryl-2*H*-1-benzopyran-2-one), also known as neoflavones, comprise a minor subclass of naturally occurring flavonoids. Because of their broad-spectrum biological activities, arylcoumarins have been attracting the attention of the organic and medicinal chemistry communities, and are considered as an important privileged scaffold. Since the development of Pechmann condensation, a classical acid-catalyzed condensation between phenol and β-keto-carboxylic acid, several versatile and efficient synthetic approaches for 4-arylcoumarins have been reported. This review summarizes recent advances in the synthesis of the 4-arylcoumarin scaffold by classifying them based on the final bond-formation type. In particular, synthetic methods executed under mild and highly efficient conditions, such as solvent-free reactions and transition metal catalysis, are highlighted.

## 1. Introduction

The Flavonoid families, a broad collection of natural products with a C_6_–C_3_–C_6_ carbon framework, exhibit diverse and potent biological activities, and hence, are valuable research tools in pharmacological and related sciences. The 15-carbon tricyclic skeleton of this family, aryl benzopyrone, is considered an important natural privileged scaffold in the fields of synthetic and medicinal chemistry [1]. Hence, various synthetic methodologies have been developed and continually improved to provide natural, as well as synthetic flavonoids in a highly efficient and atom-economical fashion.

4-Arylcoumarins (4-aryl-2*H*-1-benzopyran-2-ones), also called as neoflavones, are a relatively minor class of naturally-occurring flavonoids, as compared to isomeric flavones (2-aryl chromen-4-one) and isoflavones (3-aryl chromen-4-one). However, these compounds have also attracted a great deal of attention, and are considered important privileged structures because of their diverse biological activities and high synthetic versatility [2,3,4,5,6]. As described in a very recent review [7], their biological activities are mainly attributed to electrophilic lactone core. The electrophilic lactone moiety that gives the coumarins the ability to form strong interactions with target proteins, such as covalent bonds or hydrogen bonds, has been considered as an essential pharmacophore to express biological activities. Liphophilic parts including 4-aryl moiety can also contribute to improving their biological activities, target selectivity, or physico-chemical properties. In this connection, derivatizations of 4-arylcoumarins to identify bioactive compounds have been focused on the modification of two aryl moieties (A and B rings), rather than the 2-pyrone moiety.

**Figure 1:**
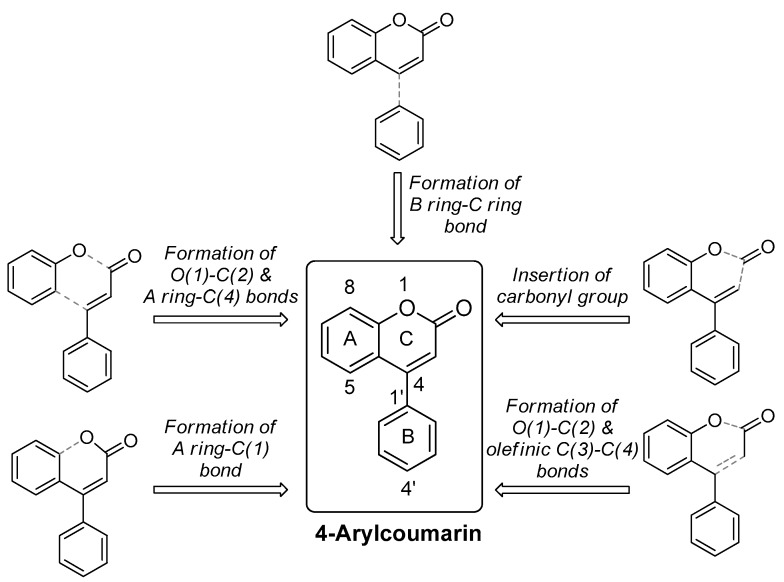
Representative synthetic routes toward 4-arylcoumarin.

While classifying synthetic routes in this review, we have focused on the final bond formation of the 4-arylcoumarin skeleton, rather than extensively covering the literature for the modification of 4-arylcoumarins and their biological activities. Generally, to synthesize the 4-arylcoumarin skeleton, a bond between the 2-pyrone nucleus (C ring) and the 4-aryl group (B ring) or bonds in the C ring have been connected at the final step. In this regard, we classified the synthetic routes on the basis of the final bond-formation type, as shown in Figure 1, and reviewed recent advancements in each case.

First, this review introduces the synthetic methods for the formation of two bonds: the O(1)−C(2) and A ring−C(4) bonds, in the C ring. Acid-catalyzed condensation between phenols and β-keto-carboxylic acid derivatives, also known as Pechmann condensation, is one of the popular approaches to furnish the 2-pyrone moiety of the 4-arylcoumarin in one pot (Section 2.1). Alternatively, Lewis acid-promoted inter- and intramolecular hydroarylation can form the two bonds of the 2-pyrone moiety when an aryl propiolate is used as synthetic equivalent for the electrophilic synthon instead of the β-keto carboxylic acid (Section 2.2). Transition-metal-catalyzed C–H functionalization and subsequent intramolecular esterification between the phenolic compound and the arylpropionate or its equivalent has also been utilized to construct the O(1)−C(2) and A ring−C(4) bonds (Section 2.3).

In addition to a summary of recent synthetic methods for the 2-pyrone moiety via the formation of O(1)−C(2) and A ring−C(4) bonds, this review provides an update of other synthetic routes toward the central 2-pyrone. Cyclocarbonylation as well as cyclocaboxylation can provide the 2-pyrone structure of 4-arylcoumarins by connecting a carbonyl synthon with the 2-vinylphenol intermediate (Section 3). A 2-hydroxybenzophenone intermediate with a two-carbon (C2 and C3) synthon can also be used to synthesize 4-arylcoumarins via esterification along with olefin formation reactions, such as Wittig-type reactions (Section 4.1) and aldol-type reactions (Section 4.2). Nucleophilic addition of an acetylide to the 2-hydroxybenzophenone intermediate, and subsequent cycloisomerization, also affords 4-arylcoumarin by forming the two aforesaid bonds (Section 4.3).

Most synthetic routes to the central 2-pyrone moiety of 4-arylcoumarins involve O(1)−C(2) ester bond formation, as mentioned above. However, bond formation between the A ring and O(1), such as intramolecular cyclization of phenylacrylic acids, can be an exceptional method for the construction of the 2-pyrone moiety (Section 5).

The formation of a bond between the C ring and the 4-aryl group (B ring) can be considered a useful synthetic strategy, especially for the late-stage diversification of bioactive 4-arylcoumarins. Moreover, transition-metal-catalyzed coupling reactions, such as Suzuki and Stille reactions, have been widely used to introduce diverse aryl groups at the 4-position of 4-halocoumarins or activated coumarin intermediates. In addition to activated coumarin intermediates, several 4*H*-coumarins and their equivalents (such as cinnamate derivatives) have been used for the synthesis of 4-arylcoumarins by transition-metal-catalyzed coupling reactions such as Heck-type reactions (Section 6.1). Several examples of 4-arylcoumarin preparation from 2-hydroxycinnamic acid derivatives via transition-metal-catalyzed coupling reactions and concurrent lactonization are also discussed (Section 6.2).

## 2. Synthetic Routes toward Central 2-pyrone of 4-arylcoumarin via O(1)–C(2) and A Ring–C(4) Bond Formation

### 2.1. Pechmann Condensation

Pechmann condensation, named after the German chemist Hans von Pechmann [8], is one of the most popular reactions for the synthesis of coumarins, including 4-arylcoumarins. This reaction starts with the condensation between a phenol and a β-keto-carboxylic acid, or its equivalent, under acidic conditions. The immense interest in the biological and medicinal importance of coumarin derivatives has triggered the development of various catalysts and reactions conditions in this regard. In addition, the mechanism undelaying the Pechmann condensation, which is still controversial, has been discussed in depth by both theoretical and experimental methods [9,10,11]. Recently, advances in the synthesis of coumarins via the Pechmann condensation have been extensively reviewed [12]. Thus, the selected syntheses of 4-arylcoumarins via the Pechmann condensation reported over the period 2000–2017 are summarized here.

Typically, the Pechmann condensation is catalyzed by a classical acid such as sulfuric acid, hydrochloric acid, hydrofluoric acid, phosphoric acid, or trifluoroacetic acid. In contrast, recently reported methods claim milder conditions which generate less toxic waste. From this point of view, the development of a solvent-free variant of the Pechmann reaction is an apparent trend toward the synthesis of 4-arylcoumarins. Establishing solvent-free reactions simplified not only the experimental procedure, but also the work-up procedure, thus saving labor and reducing pollution. In particular, the non-chromatographic purification of products, such as simple recrystallization, is in good accordance with the principle of green chemistry.

The conditions developed for the synthesis of 4-arylcoumarins through solvent-free Pechmann condensation are summarized in Table 1. Sugino and Tanaka reported a simple operation and easy work-up procedure using p-TsOH, so that wastes were minimized (Entry 1) [13]. Sharma et al. also reported the use of p-TsOH, with grinding at room temperature (Entry 2) [14]. The moisture absorbed during the grinding made the reaction mixture homogeneous, and the product could be isolated by dilution with ice-cold water. A microwave-accelerated variant of the reaction in the presence of p-TsOH has been investigated (Entry 3) [15]. Sulfamic acid [16] and meglumine sulfate [17] were found to be efficient and mild acids for the synthesis of 4-arylcoumarins (Entries 4–6). Microwave-accelerated reaction in the presence of meglumine sulfate was also compared with the corresponding thermal reaction. Efforts aimed at identifying a readily available and inexpensive organic acid revealed that trichloroacetic acid would be as an efficient catalyst (Entry 7) [18]. Selectfluor^TM^, a well-known fluorinating agent, is reported to be an efficient catalyst for the synthesis of 4-arylcoumarins as it is acidic (Entry 8) [19]. Metal halides such as ZrCl_4_ [20], VCl_3_ [21], SnCl_4_ [22], CuCl_2_, CuBr_2_ [23], BaCl_2_ [24], and LiBr [25] were investigated for use in a mild and solvent-free Pechmann condensation to provide 4-arylcoumarins (Entries 9–14). Ultrasound was found to synergistically accelerate the condensation in the presence of BiCl_3_ [26] (Entry 15). Thermal and microwave-accelerated reactions were compared in the presence of a highly efficient and recyclable catalyst, CoPy_2_Cl_2_ [27] (Entries 16, 17). Sc(OTf)_3_ [28] and Mg(NTf_2_)_2_ [29] served as efficient catalysts for the synthesis of 4-arylcoumarins under solvent-free conditions (Entry 18–19). Unlike most Lewis acids, which are deactivated in the presence of aqueous or protic solvents, Sc(OTf)_3_ and Mg(NTf_2_)_2_ are known to be stable and work well even in water. In fact, Mg(NTf_2_)_2_ recovered by a simple extraction with water catalyzed the same Pechmann condensation without significant loss of catalytic activity after several runs [29]. Pechmann condensations could also be catalyzed by Bi(NO_3_)_3_·5H_2_O [30], CAN [31], and Y(NO_3_)_3_ [32] (Entries 20–23). Among them, CAN-catalyzed reactions under thermal and microwave conditions were also compared [31]. Cu(CH_3_CN)_4_PF_6_ [33], and MnSO_4_ [34] were also found to be efficient Lewis acids for the synthesis of coumarins (Entries 24,25). Moradi and Belali reported the use of molybdate sulfuric acid in the synthesis of 4-substituted coumarins via the Pechmann condensation (Entry 26) [35]. Molybdate sulfuric acid has the advantage of being insoluble in organic solvents, making the work-up procedure easy (filtration). Goswami reported organo- and nano-cocatalytic conditions, i.e., the use of pyridine dicarboxylic acid and nanocrystalline zinc oxide, for the synthesis of coumarins via the Pechmann condensation (Entry 27) [36]. Nanosized WO_3_-ZrO_2_ particles were prepared by solution combustion synthesis using urea as the fuel and applied to the Pechmann condensation [37] (Entry 28).

As an alternative approach, Pechmann condensation using solid supports has been devised (Table 2). Silica-supported solid catalysts such as silica chloride [38], silica-supported sulfuric acid [39,40], silica gel-supported zirconyl chloride ocatahydrate [41], silica-supported boric trisulfuric anhydride [42], and SnCl_4_ grafted on silica gel [43] have been applied successfully for the synthesis of 4-arylcoumarins via the Pechmann condensation (Entry 1–6). Silica supports have the advantages of good accessibility, excellent chemical and thermal stability, and large surface area for easy functionalization. In addition, ASA [44] has been reported as the catalyst for Pechmann condensation reactions, similar to silica-supported sulfuric acid (Entry 7).

Both synthetic polymers and biopolymers have been investigated for use in the synthesis of 4-arylcoumarins. PVSA [45], an aliphatic polymeric sulfonic acid, could be easily recovered from a water filtrate after completion of the reaction, as it is highly soluble in water and lower alcohols (Entry 8). XSA [46] is based on the biodegradable polymer xanthan, which is readily available from a renewable agro resource (Entry 9). Thermal and microwave-accelerated reactions were compared in the presence of CSA (Entries 10, 11) [47].

Magnetic solid-supported catalysts were prepared and explored for the synthesis of 4-arylcoumarins. An apparent advantage of these catalysts is the ease of recovery using an external magnet. MNESA was recovered and reused in seven cycles without significant loss of activity (Entry 12) [48]. The recovered γ-Fe_2_O_3_@Hap-Ag NPs were reused in ten consecutive cycles (Entry 13) [49]. KAl(SO_4_)_2_·12H_2_O (alum) is a solid Lewis acid with mild acidity, and it is insoluble in organic solvents. Dabiri et al. and Azizian et al. independently reported the synthesis of coumarins via the Pechmann condensation in the presence of alum as a solid acid catalyst (Entry 14) [50,51].

It is noteworthy that substituents strongly affect the Pechmann condensation. Although recent advances in the development of methods for the Pechmann condensation provided milder conditions with less toxic wastes, substituents are still limited to hydroxy, alkoxy, or alkyl substituents in many cases. Thus, the issue of applying Pechmann condensation for the preparation of 4-arylcoumarins possessing various substituents by using a green condition should be resolved in the future.

### 2.2. Hydroarylation of Phenylpropiolate

Lewis acid-promoted hydroarylation of arylpropiolates is an alternative method of Pechmann condensation for the synthesis of 4-arylcoumarins. Recently, several improved versions of the reaction have been reported, as summarized in Table 3.

Solvent-free condensation reactions in the presence of a catalytic amount of indium chloride or zinc chloride have been explored (Entries 1, 2) [52,53]. H_14_P_5_NaW_30_O_110_, a heteropolyacid with a Preyssler structure, has also been investigated as an efficient catalyst (Entry 3) [54]. This catalyst is recyclable, and both air- and moisture stable.

**Scheme 1:**
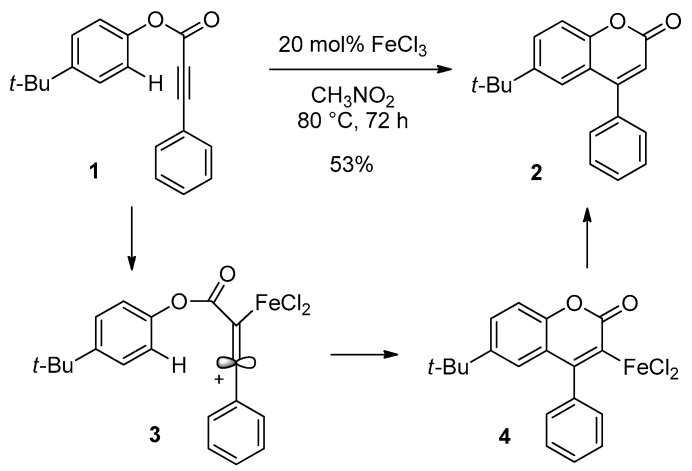
Synthesis of 4-arylcoumarins via FeCl_3_-catalyzed intramolecular alkenylation of arenes.

Li et al. developed an intramolecular alkenylation of arenes to be applied for the synthesis of 4-arylcoumarins during the course of their study on the addition of electron-rich arenes to aryl-substituted alkynes in the presence of FeCl_3_ (Entry 4) [55]. The proposed mechanism is quite different from that of some other hydroarylations that proceed via aromatic C–H activation. This is suggested to be a Friedel-Crafts-type process, which involves the formation of aryl-substituted alkenyl cation intermediate **3**, followed by electrophilic aromatic substitution, to produce intermediate **4** (Scheme 1). Kutubi et al. developed FeCl_3_/AgOTf-catalyzed cascade reactions (Entry 5) [56] involving hydroarylation, inspired by the work of Li et al., in which FeCl_3_ was used as a catalyst without additives [55].

Microwave-accelerated reactions have also been reported. Fiorito et al. a reported Yb(OTf)_3_-promoted reaction under microwave irradiation (Entry 6) [57]. Bennardi et al. reported microwave-assisted synthesis using silica-supported Wells-Dawson acid as a heterogeneous catalyst under solvent-free conditions (Entry 7) [58].

The use of ionic liquids was also reported for the synthesis of 4-arylcoumarins [59,60]. Song et al. reported hydrophobic ionic liquids that dramatically enhanced the activity of metal triflates in the intramolecular Friedel-Crafts alkenylation of aromatic compounds. The optimized conditions in a mixture of [bmim][SbF_6_] and methylcyclohexane, in the presence of Hf(OTf)_4_, were successfully extended to the synthesis of 4-phenylcoumarins [59]. Mechanistic studies confirmed that the reaction proceeds via a vinyl cationic intermediate [60].

### 2.3. Transition-Metal-Catalyzed C–H Bond Functionalization

**Scheme 2:**
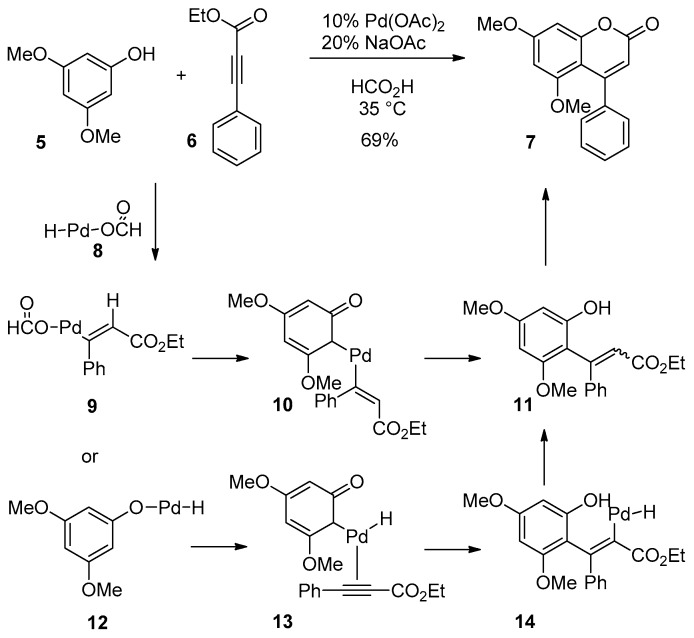
Pd-catalyzed formation of 4-arylcoumarins via C–H insertion.

Trost and Toste reported a seminal report on the atom-economic synthesis of coumarins, including 4-arylcoumarins, in the presence of palladium catalysts in formic acid (Scheme 2) [61,62]. The proposed mechanism involves the formation of palladium phenoxide **12** from hydridopalladium carboxylate **8** and phenol **5**, or alternatively, the formation of vinylpalladium intermediate **9** from **8** and alkynoate **6**, wherein Pd(0) is the precatalyst, rather than Pd(II) species.

**Scheme 3:**
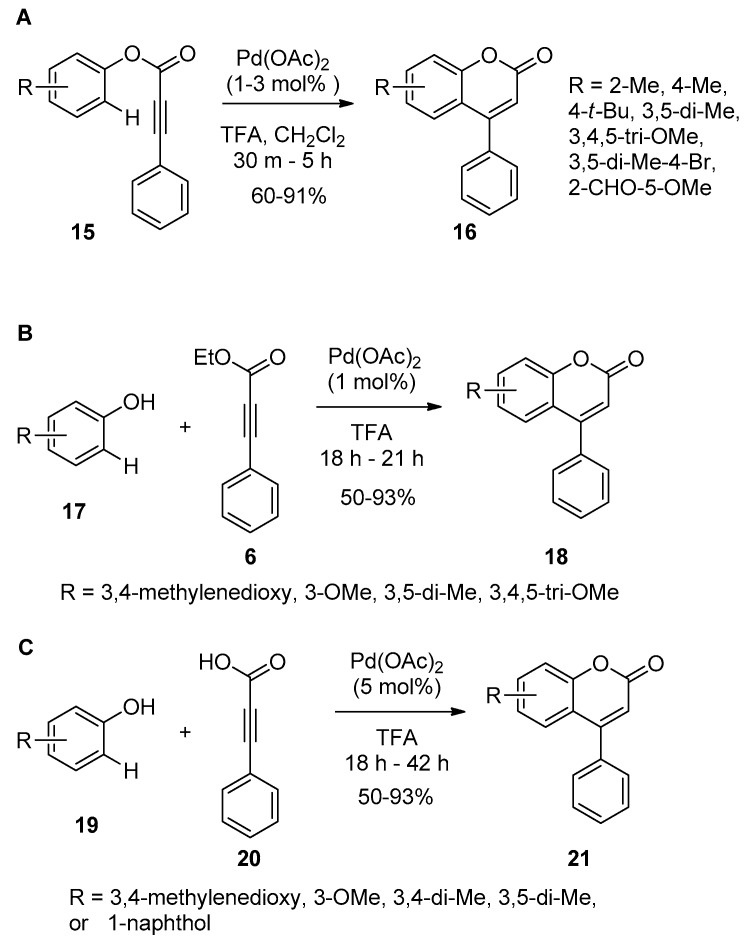
Pd-catalyzed intramolecular (**A**) and intermolecular (**B**,**C**) hydroarylation of C–C triple bonds for the preparation of 4-arylcoumarins.

Jia et al. also reported a method for hydroarylation with the activation of the C–H bond for addition to multiple C–C bonds (Scheme 3A) [63,64]. Direct functionalization of the C–H bond for the synthesis of 4-arylcoumarins **16** proceeds through electrophilic metalation of the aromatic C–H bond (**15**), leading to C–C bond formation, followed by regio- and stereoselective addition to alkynes. In this case, the requirement of a large excess of TFA as the solvent to keep the cationic Pd(II) species is noteworthy. Following this, intermolecular versions of the one-step methods were also reported (Scheme 3B,C) [65,66]. Intriguingly, Tunge and Foresee argued that the hydroarylation proceeds not via C–H bond activation, but via aromatic electrophilic substitution [67].

**Scheme 4:**
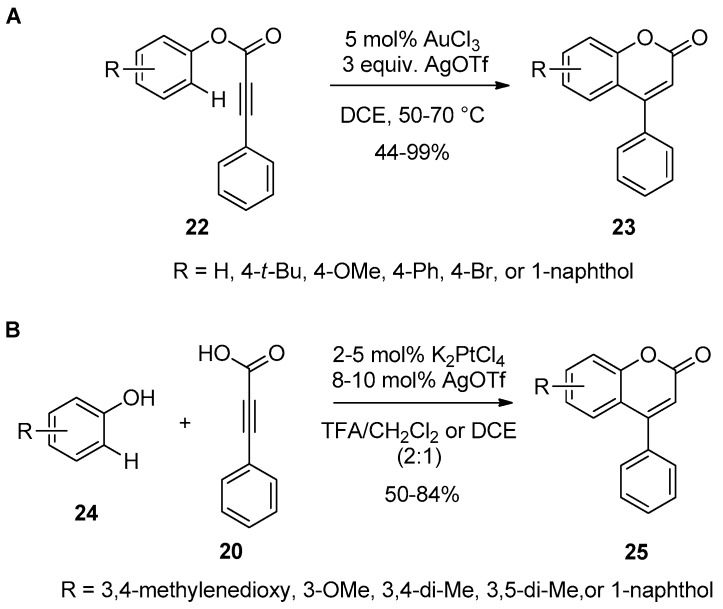
Synthesis of 4-arylcoumarins via Au (**A**)- and Pt (**B**)-catalyzed intramolecular addition.

Gold [68] and platinum [67] were also found to be efficient catalysts for the preparation of 4-arylcoumarins through C–H bond functionalization. Shi and He reported an intramolecular addition reaction for the synthesis of 4-arylcoumarin **23** in the presence of AuCl_3_ pretreated with 3 equiv. of AgOTf (Scheme 4A) [68]. The use of silver salts was suggested to help generate a more electrophilic Au(III) species. Oyamada and Kitamura also reported PtCl_2_ and K_2_PtCl_4_ as efficient catalysts in the presence of AgOTf as an additive for the synthesis of coumarin **25** (Scheme 4B) [67]. The mechanism is supposed to be similar to that for the Pd(II)-catalyzed reaction of propiolates with phenols to afford coumarins [65].

**Scheme 5:**
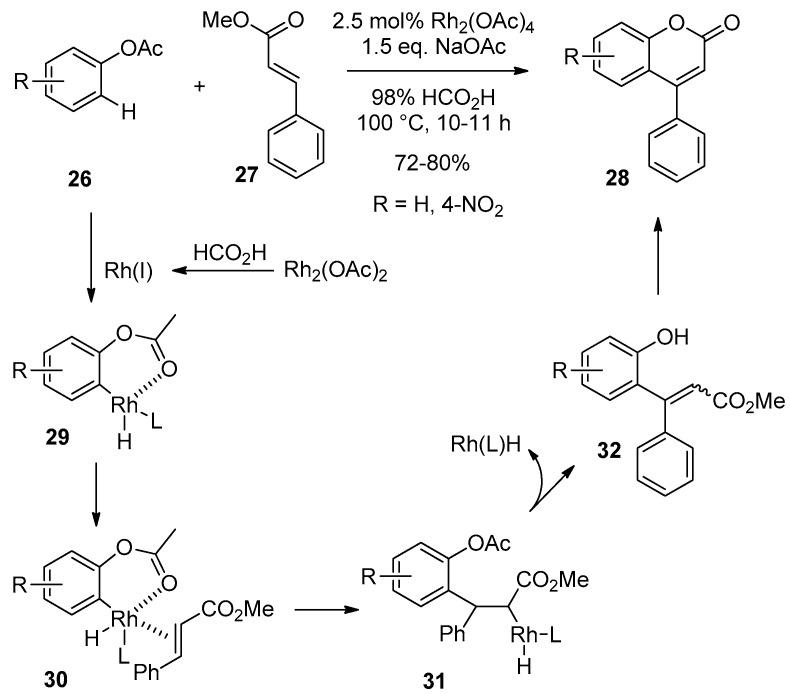
Synthesis of 4-arylcoumarins via Rh-catalyzed C–H bond activation.

Rh-catalyzed C–H bond activation has also been applied to the synthesis of 4-arylcoumarins. Gadakh et al. developed a Rh-catalyed *ortho*-C–H bond activation of phenolic acetate **26** with acrylate **27** in HCO_2_H [69]. This methodology worked well even in the presence of a strong electron-withdrawing group. HCO_2_H served as a reducing agent to produce Rh(I) species in the presence of NaOAc (Scheme 5). Ortho-metalation of phenolic acetate **26** provides rhodacycle intermediate **29**. Coordinative insertion of alkenic ester **30** gives intermediate **31**, followed by β-hydride elimination to release the alkenated product **32**. Finally, intramolecular cyclization produces coumarin **28**.

## 3. Cyclocarbonylation and Cyclocarboxylation toward Central 2-Pyrone of the 4-Arylcoumarin

**Scheme 6:**
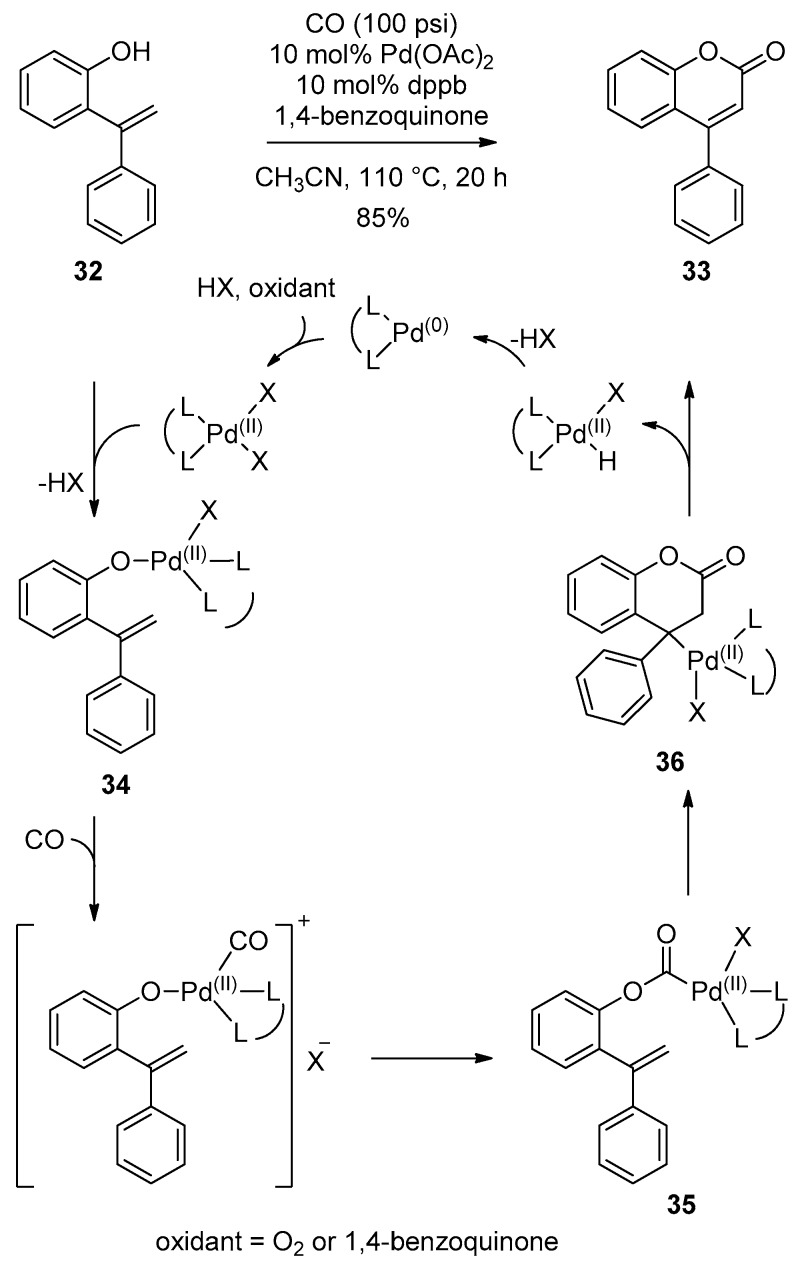
Synthesis of 4-arylcoumarins via Pd-catalyzed cyclocarbonylation.

Recently, lactonization by direct cyclocarbonylation or cyclocarboxylation has been demonstrated to be a powerful strategy for constructing the pyrone moiety of coumarins [70,71]. Cyclocarbonylation of 2-vinylphenol using a transition-metal catalyst is an atom-economical and environmentally attractive method for 4-arylcoumarin synthesis. In 2012, Alper et al. reported a novel method for the synthesis of coumarins via palladium-catalyzed intramolecular oxidative cyclocarbonylation, in which a terminal alkene was directly coupled with a phenol (Scheme 6) [72]. The Pd-catalyzed cyclocarbonylation of 2-vinylphenol **32** afforded 4-arylcoumarin **33** under 100 psi CO, with 1,4-benzoquinone as the oxidant. A possible mechanism for the Pd-catalyzed cyclocarbonylation is depicted in Scheme 6. Palladium phenoxide intermediate **34** is produced from **32** by ligand exchange. Consequently, phenoxycarbonyl palladium complex **35** is generated by CO insertion into the Pd-O bond. Thereafter, alkene insertion into the Pd-CO bond of **35** affords alkylpalladium **36**. Finally, 4-arylcoumarin **33** is formed by β-hydride elimination. The resulting Pd(II) hydride complex is reduced to a Pd(0) species, which is regenerated to the active Pd(II) catalyst by the oxidant.

**Scheme 7:**
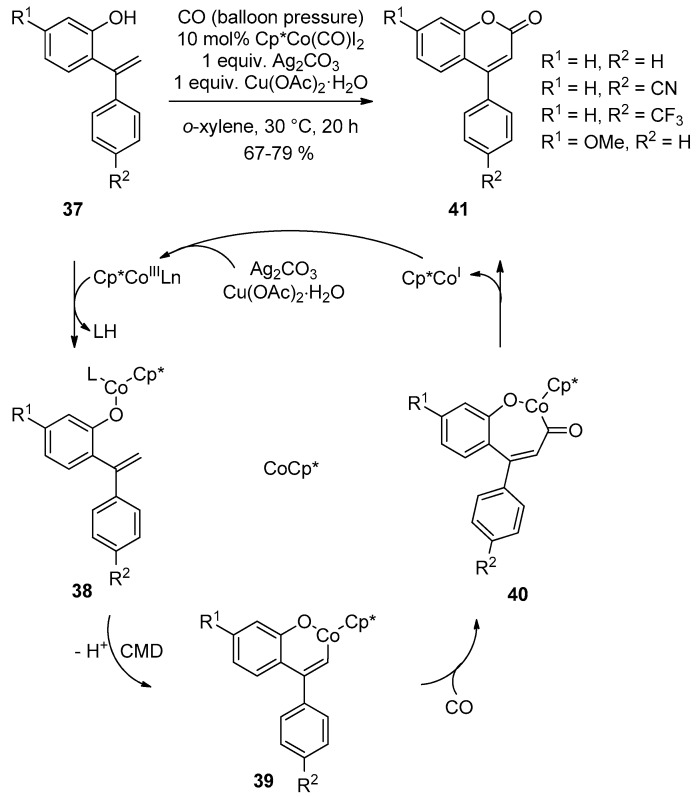
Synthesis of 4-arylcoumarins via Cp*Co(III)-catalyzed cyclocarbonylation.

More recently, Wang et al. described a rather mild cyclocarbonylation of 2-vinylphenols **37** in the presence of Cp*Co(III) catalyst (Scheme 7) [73]. With Ag_2_CO_3_ and Cu(OAc)_2_·H_2_O as co-oxidants, the reaction could sufficiently be proceeded under a balloon pressure of CO. A plausible mechanism of the reaction is depicted in Scheme 7. After the generation of the active catalyst Cp*Co(III)Ln, 2-vinylphenol **37** undergoes ligand replacement to form cobalt phenoxide intermediate **38**. Thereafter, cyclometalated species **39** might be formed via a concerted metalation deprotonation (CMD) pathway based on the kinetic isotope effect studies, not a process of an intramolecular electrophilic attack and subsequent deprotonation. CO insertion into **39** and subsequent reductive elimination of **40** afford 4-arylcoumarin **41**.

**Scheme 8:**
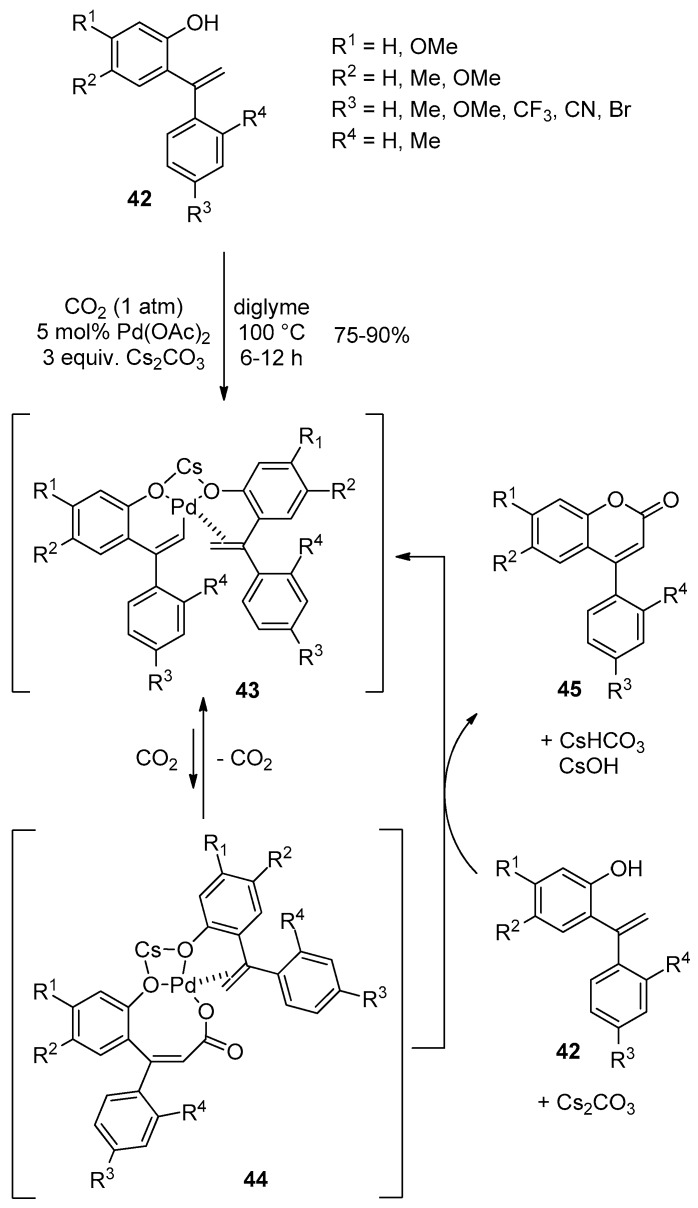
Synthesis of 4-arylcoumarins via Pd-catalyzed cyclocarboxylation.

Utilizing carbon dioxide (CO_2_) as a C1 synthon for organic synthesis has received much attention from the synthetic community, because it is a user-friendly and abundant source [74,75,76]. Hence, the direct carboxylation of alkenyl C–H bonds with CO_2_ is a highly attractive method for straightforward coumarin synthesis. In 2013, Iwasawa et al. reported 4-arylcoumarin synthesis via an unprecedented palladium-catalyzed direct carboxylation of the alkenyl C–H bond of α-phenyl-2-hydroxystyrene **42** with CO_2_ [77]. Scheme 8 shows the mechanism of the Pd-catalyzed alkenyl C–H carboxylation. Firstly, the cyclometalated intermediate **43** is formed by the coordination of Pd(OAc)_2_, Cs_2_CO_3_ and two molecules of **42** via a chelation-assisted alkenyl C–H bond cleavage. Subsequently, palladium carboxylate intermediate **44** is generated by the reversible nucleophilic carboxylation of **43**. Participation of the third **42** and the base regenerates the alkenyl palladium species, along with the formation of 4-arylcoumarin **45**. The reaction could be applied to the synthesis of various functionalized 4-arylcoumarins in good yields.

**Scheme 9:**
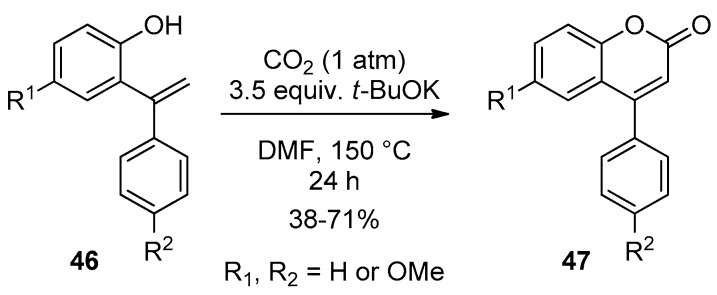
Synthesis of 4-arylcoumarins via transition-metal-free cyclocarboxylation.

In 2017, Zhi et al. reported a transition-metal-free and redox neutral lactonization by cyclocarboxylation of 2-alkenylphenol **46** to afford 4-arylcoumarin **47** [71] (Scheme 9). They stated that the yield varies depending on the para substituent on the phenol ring. When R^1^ and R^2^ were H, the product was generated in 38% yield, with the ortho- and para carboxylated byproducts of the phenol ring. However, improved yields could be obtained when R^1^ was a methoxy or methyl group.

## 4. Synthetic Routes toward Central 2-Pyrone of 4-Arylcoumarin via C(3)−C(4) Double Bond and O(1)−C(2) Ester Bond Formation

### 4.1. Wittig-Type Olefinations

**Scheme 10:**
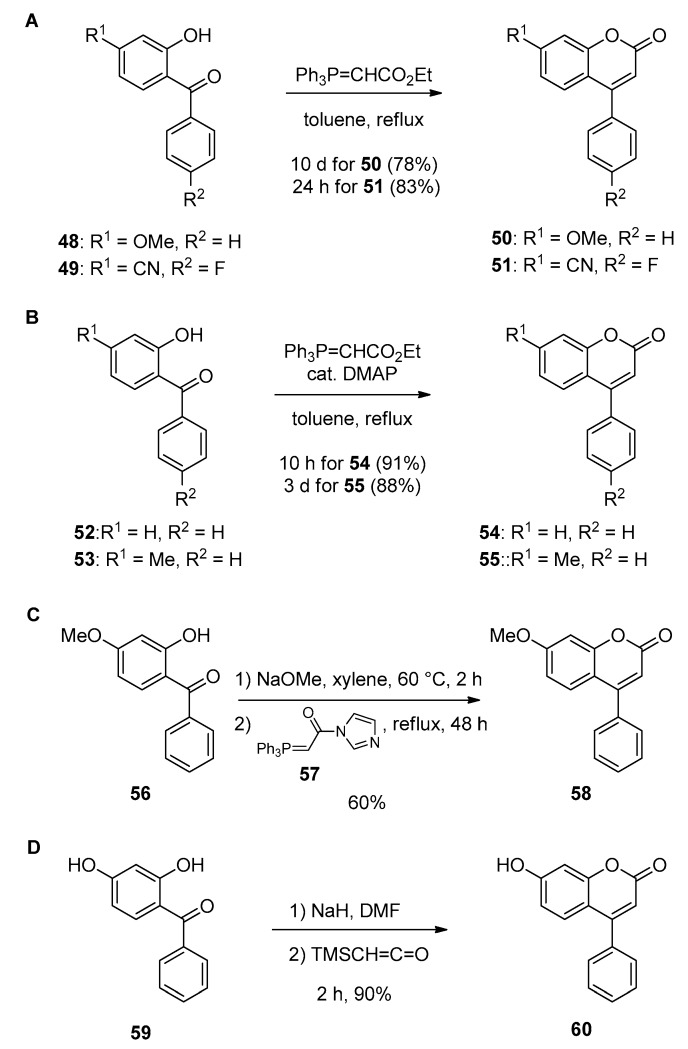
Synthesis of 4-arylcoumarins via Wittig-type reaction (**A**,**B**), intramolecular Wittig olefination (**C**), and Peterson olefination (**D**).

Wittig-type reactions, with the concurrent esterification of 2-hydroxybenzophenones, have been widely used for the synthesis of 4-arylcoumarins because of their generality and effectiveness in the formation of the C(3)–C(4) double bond and O(1)–C(2) ester bond of the 4-arylcoumarin skeleton. Lepoittevin et al. reported the preparation of 7-methoxy-4-arylcoumarin **50** from 2-hydroxybenzophenone **48** by the Wittig reaction in the synthesis of (*R*)- and (*S*)-4-methoxyalbergione [78]. In 2013, Aidhen et al. have also reported the synthesis of 4-arylcoumarin **51** as an intermediate for MK-0633 [79] under conditions similar to those reported by Lepoittevin (Scheme 10A). These one-pot synthesis for the target molecules are proceeded under reflux conditions in toluene solvent. When 2-hydroxybenzophenones were treated with the stable phosphorane ethyl-2-(triphenylphosphoranylidene)acetate, the Wittig reaction firstly occurred to form α,β-unsaturated esters. Consequently, an intramolecular lactonization provided functionalized 4-arylcoumarines. Lipshutz et al. and Cui et al. demonstrated the synthesis of 4-arylcoumarins via a Wittig-type reaction using DMAP as the catalyst [80,81], which possibly promoted the lactonization (Scheme 10B). In 2009, Kumar et al. reported an interesting 4-arylcoumarin synthesis via esterification and subsequent intramolecular Wittig cyclization (Scheme 10C) [82]. 2-Hydroxybenzophenone **56** was first esterified with ylide **57**, which was prepared by using carbonyl diimidazole and methylenetriphenylphosphorane. Then, the corresponding phosphorane intermediate underwent intramolecular Wittig cyclization to afford 4-arylcoumarin **58**. Taylor et al. described that 4-arylcoumarin **60** could be obtained from 2-hydroxybenzophenone **59** through Peterson olefination. The method gave a higher product yield in a short time, plausibly because of the formation of a phenoxide intermediate [83] (Scheme 10D).

### 4.2. Aldol-Type Olefinations

**Scheme 11:**
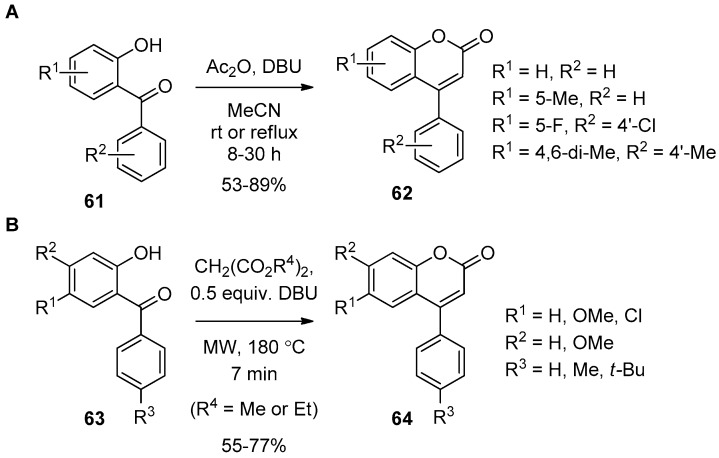
Synthesis of 4-arylcoumarins via Kostanecki-Robinson reaction (**A**) and Knoevenagel condensation (**B**).

The conversion of 2-hydroxybenzophenones to 4-arylcoumarins can be achieved by aldol-type olefination, such as the Kostanecki-Robinson reaction and Knoevenagel condensation, with subsequent lactonization. The Kostanecki-Robinson reaction has been used for coumarin [84,85] or chromone [86] synthesis by *O*-acylation of 2-hydroxyarylketones with an aliphatic anhydride, followed by aldol condensation. In 2011, Lee et al. published a synthesis method for 4-arylcoumarins **62** based on the DBU-mediated Kostanecki reaction [87] (Scheme 11A). A previous approach to 4-arylcoumarins by the Kostanecki-Robinson reaction had several drawbacks, including harsh reaction conditions and low yields. However, the DBU-mediated Kostanecki-Robinson reaction of **61** in acetonitrile proceeded at room temperature and gave the product in moderate yield. In 2011, Seijas et al. introduced a solvent-free synthesis of 4-arylcoumarins via the Knoevenagel condensation [88] (Scheme 11B). 2-Hydroxybenzophenones **63** underwent microwave-promoted Knoevenagel condensation with ethyl malonate or methyl malonate, followed by lactonization. Decarboxylation of the subsequent intermediate led to the construction of 4-arylcoumarins **64**. However, the existence of a second alcohol in the phenol ring furnished *O*-methylated or *O*-ethylated byproducts depending on the alkylmalonate.

### 4.3. Cycloisomerization

**Scheme 12:**
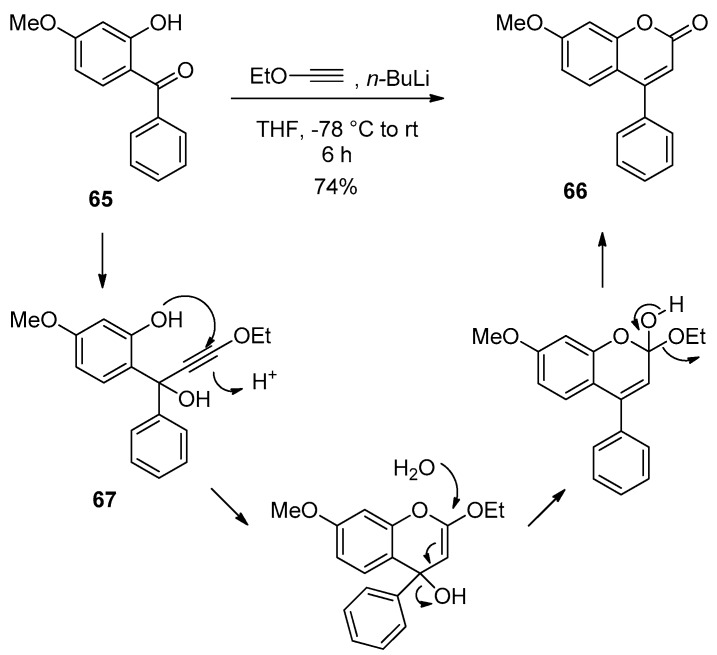
Synthesis of 4-arylcoumarins via cycloisomerization.

In 2012, Reddy et al. proposed an interesting method for the formation of the C(3)–C(4) double bond and O(1)–C(2) ester bond of 4-arylcoumarins by acetylide addition and subsequent cycloisomerization [89] (Scheme 12). Treatment of 2-hydroxybenzophenone **65** with ethoxyacetylide provided propargyl alcohol intermediate **67**. Subsequent quenching with ammonium chloride induced 6-endo selective cycloisomerization of **67** and a series of reactions to afford 4-arylcoumarin **66**. It is notable that transition-metal catalysts were not required in the corresponding reaction, unlike the conventional cycloisomerization of alkynes to prepare heterocyclic compounds.

## 5. Oxidative Cyclization toward Central 2-Pyrone of the 4-Arylcoumarin by A Ring−O(1) Bond Formation

**Scheme 13:**
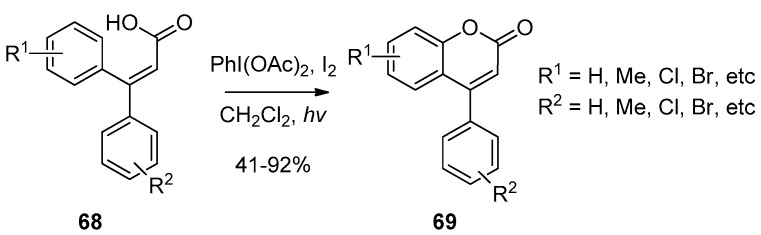
Synthesis of 4-arylcoumarins by oxidative cyclization.

Most of the reported syntheses of 4-arylcoumarins require phenol derivatives as the starting materials, indicating that methodologies featured with intermolecular linking of aryl groups (A ring) and carboxylic acids are scarcely reported. In 2013, Li et al. reported the synthesis of 4-arylcoumarins **69** via phenyliodine diacetate (PIDA)/I_2_-mediated oxidative cyclization of substituted phenylacrylic acids **68**; in other words, this reaction facilitates intermolecular assembly between aryl groups and carboxylic acids [90] (Scheme 13).

## 6. Synthesis of 4-Arylcoumarins by Introduction of An Aryl group at the 4-Position

### 6.1. Transition-Metal-Catalyzed Introduction of 4-Aryl Group

**Scheme 14:**
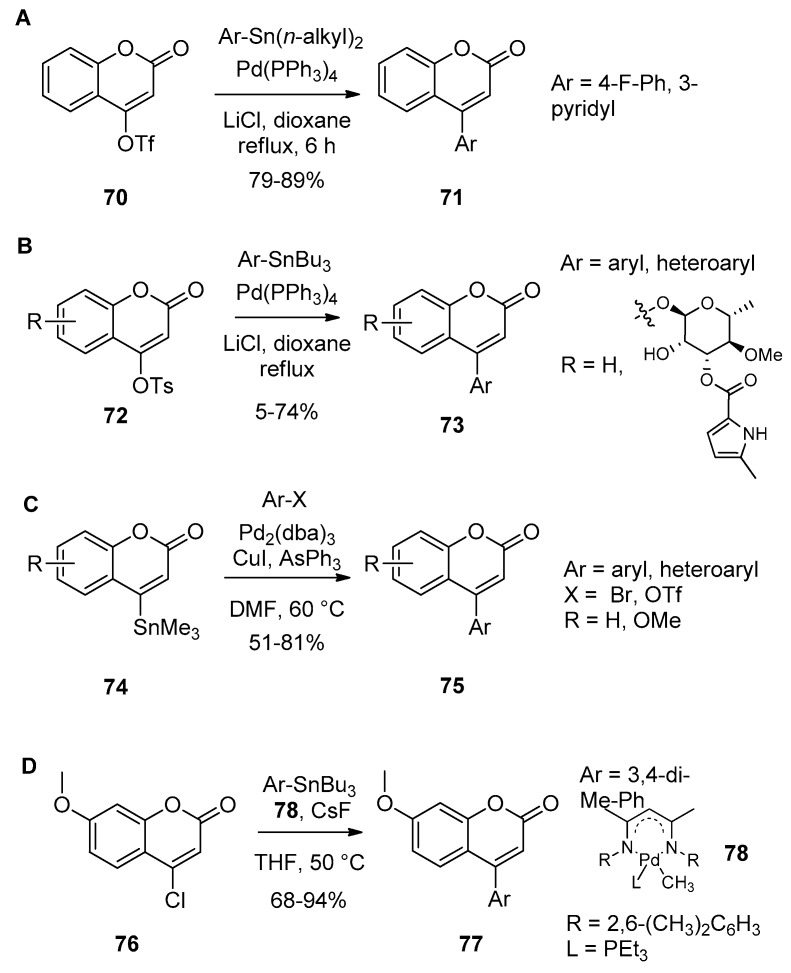
Synthesis of 4-arylcoumarins via Pd(0)-catalyzed Stille reaction using 4-triflated coumatins (**A**), 4-tosylated coumarins (**B**), 4-stannylcoumarins (**C**) and 4-chlorocoumarin (**D**).

Recently, numerous synthetic approaches for 4-arylcoumarins via the direct functionalization of coumarin intermediates have been reported. These methodologies mainly involve transition-metal-catalyzed reactions, and are widely applicable to the synthesis of natural or synthetic 4-arylcoumarins, partly because they are suitable for the introduction of various substituents on the 4-aryl moieties. Initially, Pd(0) species were used as the transition-metal catalysts in these reactions. In 1988, Wattanasin first demonstrated the feasibility of Pd(0)-catalyzed Stille coupling for the synthesis of 4-arylcoumarins **71** from 4-triflated coumarin **70** and aryl stannane species [91] (Scheme 14A). Following this report, another version of Stille coupling for the synthesis of 4-arylcoumarins **73** using 4-tosylated coumarins **72** instead of 4-triflated coumarins was reported; this reaction allowed easy access to highly functionalized 4-arylcoumarins [92] (Scheme 14B). Ciattini et al. reported a similar route to various 4-arylcoumarins **75** using 4-stannylcoumarins **74**, which is the reverse version of the aforementioned reactions [93] (Scheme 14C). More recently, it was reported that 4-chlorocoumarin **76** underwent Stille coupling with arylstannyl species, in the presence of a highly active palladium catalyst **78**, to afford 4-arylcoumarin **77** [94] (Scheme 14D).

**Scheme 15:**
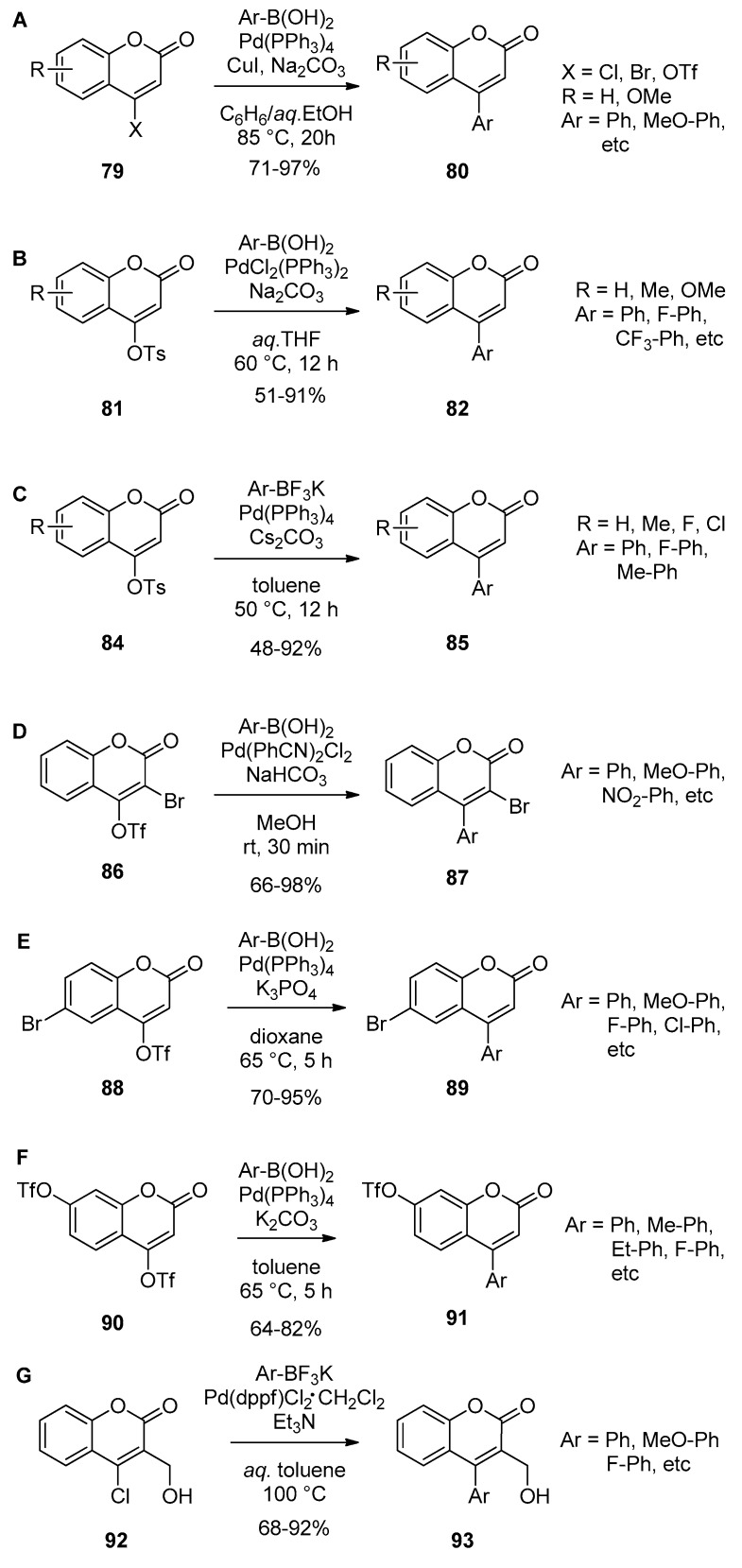
Synthesis of 4-arylcoumarins via Pd(0)-catalyzed Suzuki reaction using 4-halocoumatins (**A**), 4-tosylated coumarins (**B**,**C**), 4-triflated coumarins (**D**–**F**) and 4-chlorocoumarin (**G**).

In 1996, a Pd(0)-catalyzed Suzuki reaction for the synthesis of 4-arylcoumarins **80** using 4-halocoumarin **79** and aryl boronic acids was reported [95] (Scheme 15A). This methodology yielded diverse 4-arylcoumarins in moderate to good yields, and it has been used in the synthesis of various 4-arylcoumarins and their fluorescent derivatives [96]. Wu et al. showed that 4-tosylated coumarins **81** could be used as manageable synthetic equivalents of 4-triflated coumarins for the coupling reaction with aryl boronic acids (Scheme 15B), similar to the Stille reaction for 4-arylcoumarins [3]. In the case of using aryl trifluoroborates, instead of aryl boronic acids, the Suzuki coupling reaction was also found to be working in another report [97] (Scheme 15C). Zhang and his colleagues reported the synthesis of diverse 4-arylcoumarins **87** via site-specific arylation to the 4-carbon linked with triflate group despite the presence of a 3-bromo group in coumarins **86** [98] (Scheme 15D). Such chemoselectivity, i.e., preference for the trifloxy group over the bromo group on the coumarin skeletons, was also observed by Langer and his colleagues, who reported regioselective arylation to the 4-carbon linked with triflate group, rather than the 7-triflate group on the coumarin skeleton [99,100] (Scheme 15E,F). Recently, the Suzuki reaction was used in the synthesis of 3-hydroxymethyl-4-arylcoumarins **93**, in an effort to identify 4-arylcoumarins with biological activities [101] (Scheme 15G).

**Scheme 16:**
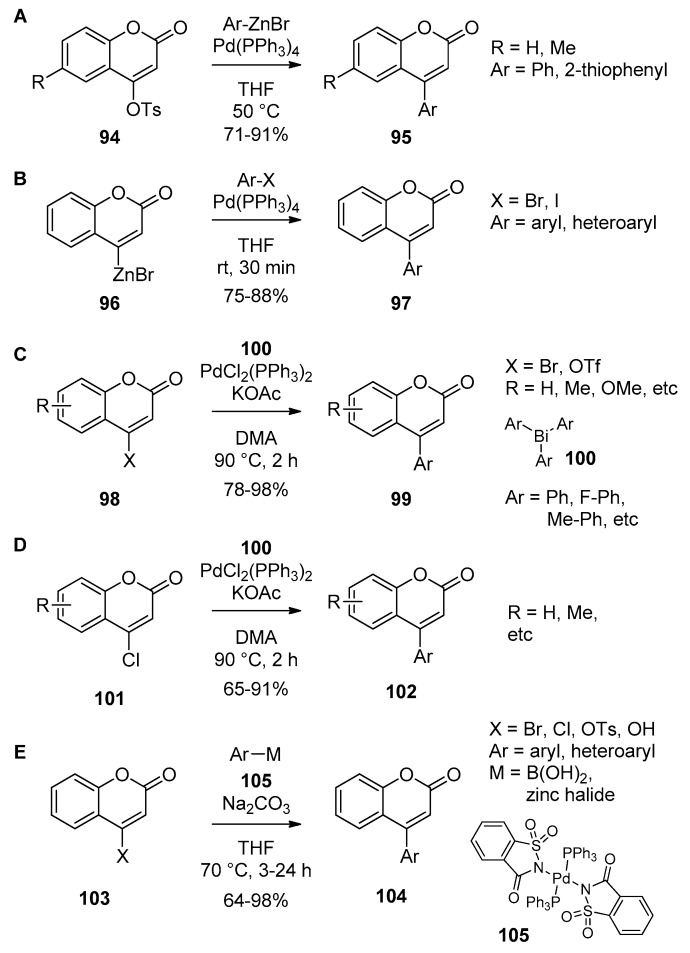
Pd(0)-catalyzed introduction of the 4-aryl group via Negishi-type reactions (**A**,**B**) and another-type reactions (**C**–**E**).

In 2001, another Pd(0)-catalysis involving the use of organozinc reagents as synthetic equivalents for the 4-aryl synthons, known as the Negishi-type coupling reaction, was reported by Wu et al. In this methodology, 4-tosylated coumarins were also used, indicating that they can be used in most variants of Pd(0)-catalyzed cross-coupling reactions for the synthesis of 4-arylcoumarins [102] (Scheme 16A). Rieke et al. reported the synthesis of 4-arylcoumarins **97** by the Negishi-type reaction of 4-coumarinylzinc bromide **96** with aryl halides [103] (Scheme 16B). Recently, Rao et al. reported triarylbismuth compounds **100** as synthetic equivalents for the 4-aryl synthon [104] (Scheme 16C). In 2013, Rao’s group also reported a similar approach for the successful preparation of 4-aryl coumarins **102** by using 4-chlorocoumarins **101** [105] (Scheme 16D). Indian researchers found that Pd(PPh_3_)_2_(saccharinate)_2_ **105** could be used as a general catalyst in both Suzuki and Negishi coupling reactions for the synthesis of 4-arylcoumains **104** [106] (Scheme 16E).

**Scheme 17:**
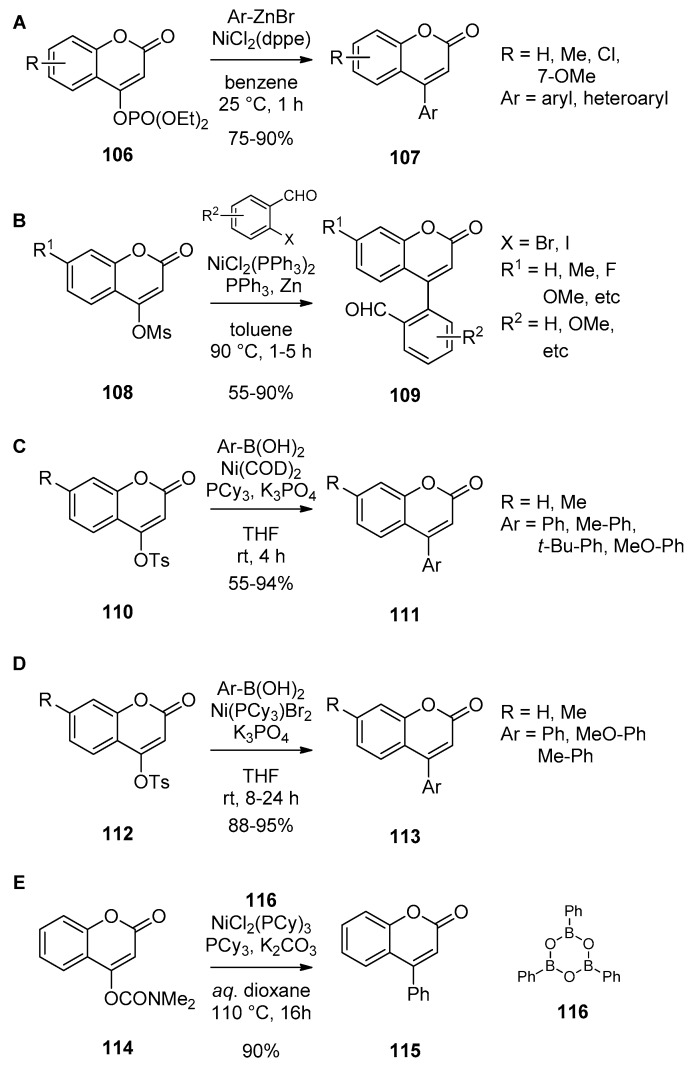
Ni(II)-catalyzed cross-coupling reactions for 4-arylcoumarin synthesis using 4-diethylphosphonooxycoumarins (**A**), 4-mesylated coumarins (**B**), 4-tosylated coumarins (**C**,**D**) and 4-carbamoyloxycoumarin (**E**).

Ni(II)-catalyzed cross-coupling reactions have been also used for the synthesis of 4-arylcoumarins. In 2001, Wu et al. first reported that 4-diethylphosphonooxycoumarins **106** and aryl zinc agents could be used as synthetic equivalents for the cross-coupling reaction toward 4-arylcoumarins [107] (Scheme 17A). Other Chinese researchers reported that a Ni(II)-catalyzed cross-coupling reaction of 4-mesylated coumarins **108** with aryl halides also successfully yielded the desired 4-arylcoumarins **109** [108] (Scheme 17B). Hu et al. found that a Ni(II)-catalyzed Suzuki reaction of 4-tosylated coumarins **110** with aryl boronic acids provided the corresponding 4-arylcoumarins **111**, especially at room temperature [109] (Scheme 17C). They also developed a more air-stable catalytic system, including Ni(PCy_3_)_2_Br_2_ for the synthesis, and reported the results elsewhere [110] (Scheme 17D). Recently, another Ni(II)-catalyzed synthetic route to 4-arylcoumarin **115** using 4-carbamoyloxycoumarin **114** and phenyl boroxin **116** was reported [111] (Scheme 17E).

**Scheme 18:**
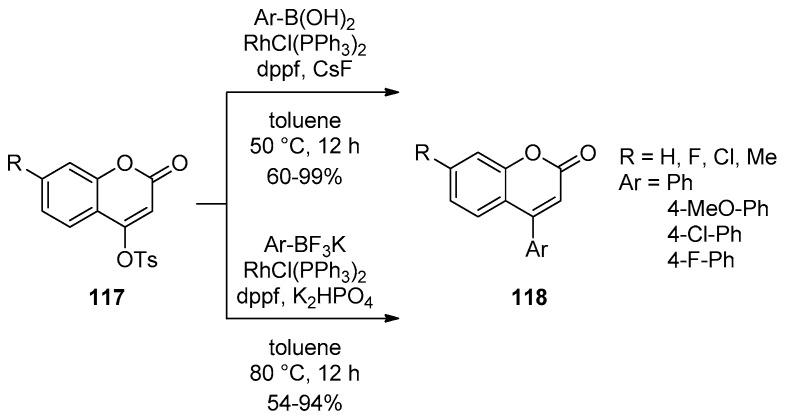
Synthesis of 4-arylcoumarins via Rh(I)-catalyzed cross-coupling reaction.

In 2006, Wu et al. reported several strategies for the Rh(I)-catalyzed Suzuki cross-coupling reaction toward the synthesis of 4-arylcoumarins. They successfully synthesized the desired coumarins by using aryl boronic acids [112] and potassium aryltrifluoroborates [113] (Scheme 18).

**Scheme 19:**
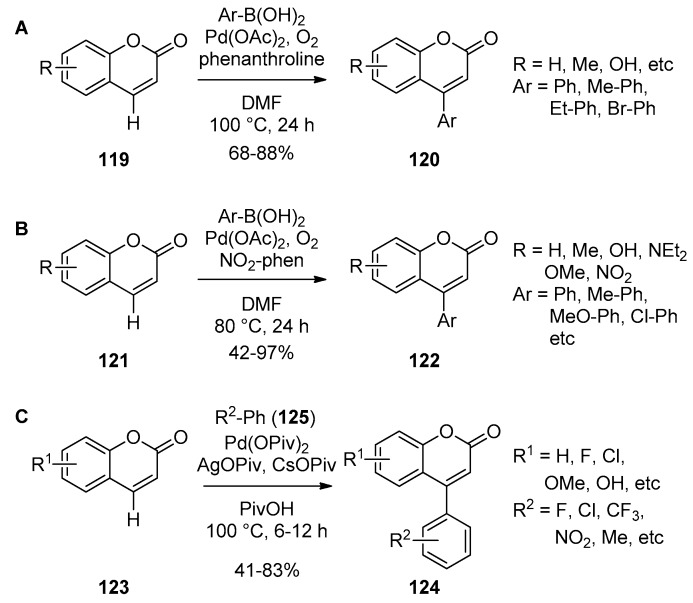
Synthesis of 4-arylcoumarins via oxidative Heck coupling reaction of the 4*H*-coumarin with aryl boronic acids (**A**,**B**) and simple arenes (**C**).

All of the above-mentioned reactions require 4-halocoumarins or other activated coumarins, and involve transition metal-catalyzed coupling with synthetic equivalents of the 4-aryl synthon for the preparation of the corresponding 4-arylcoumarins. Recently, simple coumarins that do not have halo or metal species at the 4-position were reported to be successfully transformed into 4-arylcoumarins via a Pd(II)-catalyzed oxidative Heck coupling reaction. In 2012, Khoobi et al. reported that the coupling reaction of 4*H*-coumarins **119** with aryl boronic acids furnished the corresponding 4-arylcoumarins **120** in good yields [114] (Scheme 19A). Li et al. concurrently reported a similar but independent work using nitrophenanthroline as a ligand [115] (Scheme 19B). More recently, Min et al. reported a novel and regioselective Pd(II)-catalyzed coupling reaction of 4*H*-coumarins **123** with simple arenes, involving C–H activation subject to an oxidative Heck coupling reaction [116,117] (Scheme 19C).

**Scheme 20:**
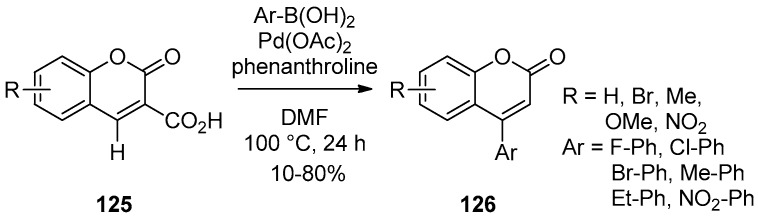
Synthesis of 4-arylcoumarins via protodecarboxylation-induced Heck reaction.

Regioselective arylation at the 4-position of coumarin was also reported by Khoobi et al., who used coumarin-3-carboxylic acids and coumarins as the starting materials [118]. They also proposed the plausible mechanism of the reaction, which mainly involved protodecarboxylation and Pd(II)-catalyzed oxidative Heck coupling (Scheme 20).

### 6.2. Domino Reactions Assisted by Transition Metal Catalysis

**Scheme 21:**
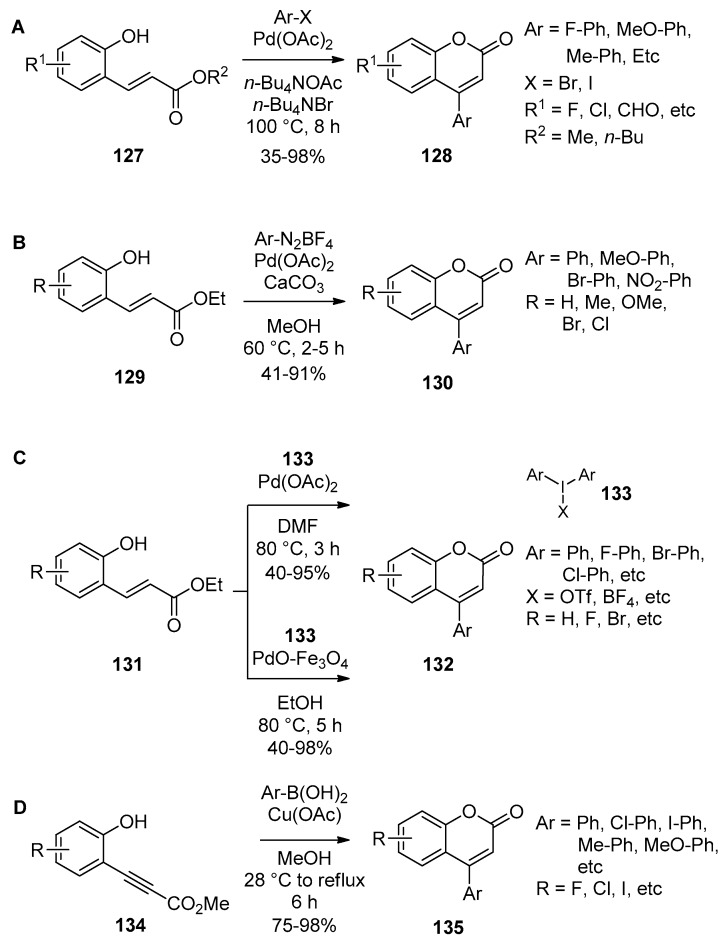
Introduction of 4-aryl group via sequential domino reactions involving Pd(II)-catalyzed oxidative Heck-type arylation (**A**–**C**) and Cu(I)-catalyzed 1,4-addition (**D**).

Recently, domino reactions initiated and assisted by transition-metal catalysis were reported for 4-arylcoumarin synthesis. These methodologies are based on cascade reactions involving the Pd(II)-catalyzed oxidative Heck-type arylation of 2-hydroxycinnamate derivatives, followed by concurrent cyclization. Thus, these approaches could be efficient tools for 4-arylcoumarin synthesis from 2-hydroxycinnamic acid derivatives by the introduction of a 4-aryl groups. In 2005, Cacchi et al. reported the first domino reaction for the synthesis of 4-arylcoumarin **128** using aryl halides as the 4-aryl sources in a molten *n*-Bu_4_NOAc/*n*-Bu_4_NBr mixture via a Heck reaction/cyclization sequence of 2-hydroxycinnamate **127** [119] (Scheme 21A). A similar approach toward 4-arylcoumarin **130** using aryldiazo compounds as the 4-aryl sources was also reported and applied for the total synthesis of (*R*)-toleterodine [120] (Scheme 21B). Wang et al. reported that diaryliodonium(III) salts **133** could be used as synthetic equivalents for the 4-aryl synthon in domino reactions for the synthesis of 4-arylcoumarin **132** [121]. In 2016, a method using Pd(II) oxide impregnated on magnetite as the catalyst (heterogenous palladium catalyst) was reported by an independent group for the synthesis of **132** via a Heck-arylation/cyclization process [122] (Scheme 21C). Another domino reaction involving the Cu(I)-catalyzed 1,4-addition of an arylboronic acid to alkyne **134** and sequential cyclization was also reported by Yamamoto and his colleague [123] (Scheme 21D).

## 7. Conclusions

4-Arylcoumarins are considered biologically-important scaffolds for the development of bioactive probes and in drug discovery. Since the Pechmann condensation was first utilized for the synthesis of 4-arylcoumarins, various synthetic routes have been developed to improve the yields, efficiency, and scope of the reaction. This review discusses recent advances in the synthetic methods for 4-arylcoumarins by classifying them based on the final bond-formation type. We hope that this review will be helpful for readers who are interested in developing novel bioactive 4-arylcoumarins and new related synthetic methods.

## Figures and Tables

**Table 1:**
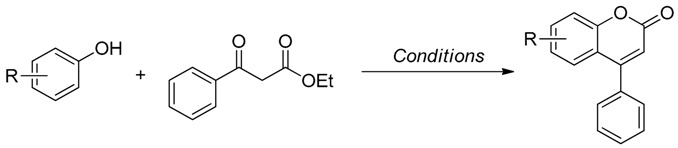
Solvent-free Pechmann condensation for the synthesis of 4-arylcoumarins.

Entry	R	Catalyst (mol%)	Temp. (°C)	Time	Yield (%)
1 [13]	3-OH	*p*-TsOH (5)	60	10 min	92
2 [14]	3-OH, 2,3-di-OH, 3,5-di-OH	*p*-TsOH (200)	rt	10 + 80 min ^a^	82–90
3 [15]	3-OH, 2,3-di-OH	*p*-TsOH (5)	MW ^b^	30s	82–92
4 [16]	3-OH, 3,5-di-OH, 2-Me-3-OH	H_2_NSO_3_H (50)	130	40–45 min	40–45
5 [17]	3-OH, 2,3-di-OH, 3,5-di-OH	Megluminesulfate ^c^	100	45–50 min	88–90
6 [17]	3-OH, 2,3-di-OH, 3,5-di-OH	Megluminesulfate ^c^	MW ^d^	6–7 min	88–92
7 [18]	3-OH, 3,5-di-OH	Cl_3_CCO_2_H (30)	100	90 min	79
8 [19]	3-OH, 3,5-di-OH, 2-Me-3-OH	Selectfluor^TM^ (50)	120	30–45 min	85–93
9 [20]	3-OH, 2,3-di-OH, 3,5-di-OH, 2-Me-3-OH	ZrCl_4_ (10)	rt	10 min	90–94
10 [21]	3-OH, 3,5-di-OH, 2-Me-3-OH	VCl_3_ (10)	50–55	2 h	84–92
11 [22]	2-OH, 3-OH, 2,3-di-OH, 3,5-di-OH, 2-Me-3-OH	SnCl_4_·5H_2_O (5)	25	5–15 min	86–94
12 [23]	3-OH, 2,3-di-OH, 3,5-di-OH, 3-Me-5-OH	CuCl_2_ or CuBr_2_ (10)	80	10–20 min	80–97
13 [24]	3-OH, 3-Me-5-OH	BaCl_2_ (10)	100	50–55 min	80–85
14 [25]	3-OH, 2,3-di-OH, 3,5-di-OH	LiBr (10)	75	15–30 min	78–86
15 [26]	3,5-di-OH, 3-Me-5-OH	BiCl_3_ (20)	US ^e^	35–40 min	76–78
16 [27]	3-OH	CoPy_2_Cl_2_ (1)	rt	3 h	92
17 [27]	3-OH	CoPy_2_Cl_2_ (1)	MW ^f^	2.5 min	96
18 [28]	3,5-di-OH	Sc(OTf)_3_ (10)	80	2 h	89
19 [29]	H, 3-OH, 3,5-di-OH	Mg(NTf_2_)_2_ (1)	80	35–60 min	85–96
20 [30]	3-OH, 3,5-di-OH	Bi(NO_3_)_3_·5H_2_O (5)	80	30 min	78–88
21 [31]	3-OH, 2,3-di-OH, 2-Me-3-OH, 3,5-di-OH	CAN	110	10–15 min	92–96
22 [31]	3-OH, 2,3-di-OH, 2-Me-3-OH, 3,5-di-OH	CAN	MW ^g^	3 min	94–97
23 [32]	3,5-di-OH, 3-Me-5-OH	Y(NO_3_)_3_·6H_2_O (10)	90	45–70 min	80
24 [33]	3-OH	Cu(CH_3_CN)_4_PF_6_ (10)	rt	20 min	82
25 [34]	3-OH, 3-OMe, 3-Me-5-OH	MnSO_4_·H_2_O (20)	100	50–100 min	75–90
26 [35]	3-OH, 2,3-di-OH, 3,5-di-OH	MSA ^g^(5)	100	40–45 min	94–95
27 [36]	3-OH, 3-OMe, 3,5-di-OH, 3,5-di-Me, 1-naphthol	pyridine dicarboxylic acid (5) + ZnO (5)	reflux	4–7 h	76, 85–88
28 [37]	2-OH, 3-OH, 2,3-OH, 1-naphthol	WO_3_-ZrO_2_^h^	MW ^i^	90–150 s	82–90

^a^ Grinding time + time for which the reaction mixture was maintained at rt. ^b^ Irradiated for 30 s at 400 W in a microwave oven. ^c^ 16 mg/mmol. ^d^ Microwave irradiated at 60–70 °C with a power of 60 W. ^e^ Irradiated in an ultrasound bath (33–35 kHz, 85 W). ^f^ The reaction mixture was placed in a microwave oven on a silica gel solid support and irradiated at 300 W. ^g^ Molybdate sulfuric acid. ^h^ 10 wt% WO3 in WO3-ZrO2, 100 mg/mmol. ^i^ Exposed to microwaves for successive irradiation of 30 s each with a cooling and mixing interval of 30 s.

**Table 2:**
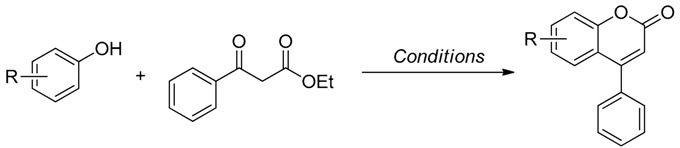
Solvent-free Pechmann condensation with solid-supported catalysis.

Entry	R	Catalyst (mol%)	Temp. (°C)	Time	Yield (%)
1 [38]	H, 3-OH, 3,5-di-OH, 3-OMe	SiO_2_-Cl ^a^	80	1–3 h	67–93
2 [40]	3-OH, 2,3-di-OH	H_2_SO_4_·SiO_2_^b^	MW ^c^	5–7 min	80–84
3 [39]	3-OH, 2,3-di-OH, 3,5-di-OH	H_2_SO_4_·SiO_2_^d^	rt	5 + 10 min ^e^	80–90
4 [41]	3-OH, 3,5-di-OH, 3-methyl-5-OH	ZrOCl_2_·8H_2_O/SiO_2_ (10)	90	40–80 min	80–94
5 [42]	2,3-di-OH, 3,5-di-OH	BTSA·SiO_2_^f^ (40)	85	1–25 min	89–90
6 [43]	3-OH, 2,3-di-OH, 3,5-di-OH, 2-Me-3-OH	SnCl_x_-SiO_2_^g^ (5)	120	3–5 h	84–91
7 [44]	3-OH	ASA ^h^	100	140 min	91
8 [45]	3-OH	PVSA ^i^ (10)	rt	25 h	91
9 [46]	3-OH	XSA ^j^	rt	20 min	96
10 [47]	3-OH	CSA ^k^	rt	20 min	96
11 [47]	3-OH	CSA ^k^	MW ^l^	2 min	97
12 [48]	2,3-di-OH, 3,5-di-OH, 3-methyl-5-OH	MNESA ^m^ (0.3)	120	3–6 h	66–71
13 [49]	3-OH, 2,3-di-OH, 3,5-di-OH, 2-Me-3-OH	γ-Fe_2_O_3_@HAp-Ag NPs ^n^	80	30–42 min	85–96
14 [50]	3-OH, 3,5-di-OH, 2-Me-3-OH	Alum ^o^ (40)	80	2–2.5 h	90–95

^a^ 200 mg/mmol, SiO_2_-Cl was prepared by refluxing thionyl chloride in the presence of a silica gel. ^b^ 100 mg/mmol. ^c^ Irradiated at 180 W in a microwave oven. ^d^ 100 mg/mmol. ^e^ Grinding time + time for which the reaction mixture was kept at rt. ^f^ Silica supported boric trisulfuric anhydride. ^g^ SnCl_4_ grafted on silica gel. ^h^ Alumina sulfuric acid, 20 mg/mmol. ^i^ Polyvinyl sulfonic acid. ^j^ Xanthan sulfuric acid, 80 mg/mmol. ^k^ Cellulose sulfuric acid, 100 mg/mmol. ^l^ The mixture was placed in a microwave oven on a silica gel solid support and irradiated at 300 W. ^m^ Fe_3_O_4_@SiO_2_@Et-PhSO_3_H = Ethane sulfonic acid organic-inorganic hybrid nanocomposite based on Fe_3_O_4_@SiO_2_ coreshell magnetic nanoparticles. ^n^ Ag supported on hydroxyapatite-core–shell magnetic γ-Fe_2_O_3_ nanoparticles. ^o^ KAl(SO_4_)_2_**·**12H_2_O.

**Table 3:**
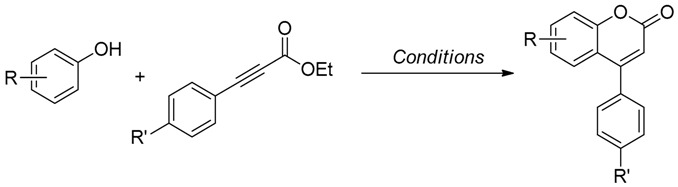
Synthesis of 4-arylcoumarins via hydroarylation of phenylpropiolate.

Entry	R	R′	Cat. (mol%)	Solvent	Temp (°C)	Time	Yield (%)
1 [52]	3-OH, 3,5-di-OH, 3,4-methylenedioxy	H	InCl_3_(~12)	Free	90	2 h	21–55
2 [53]	3-OH, 3,5-di-OH, 3,5-di-OMe	H	ZnCl_2_ (5)	Free	100	5 min–12 h	54–95
3 [54]	H, 3-OH, 3-OMe, 4-Me, 4-OMe,	H, Me, OMe	H_14_P_5_NaW_30_O_110_ (0.5)	Free	130	2 h	66–90
4 [55]	4-*t-*Bu ^a^	H	FeCl_3_ (20)	CH_3_NO_2_	80	72 h	53
5 [56]	3-OMe, 3,5-di-Me, 1-naphthol	H	FeCl_3_ (20) + AgOTf (60) + TFA ^b^	DCE	60	15 h	75–80
6 [57]	H, 2-F, 3-NO_2_, 4-Cl	H	Yb(OTf)_3_ (10)	Free	MW ^c^	2 min	91–98
7 [58]	H, 3-OH, 3,5-di-OH	H	WD/SiO_2_^d^	Free	MW ^e^	10 min	54–99

^a^ The substrate is 4′-*tert*-butylphenyl phenylpropiolate prepared by the esterification of 4-*tert*-butylphenol and phenylpropiolic acid. ^b^ 0.25–0.5 mL/mmol. ^c^ Microwave irradiated with a power of 200 W. ^d^ Silica-supported Wells-Dawson heteropolyacid (H_6_P_2_W_18_O_62_·24H_2_O). ^e^ Microwave irradiated with a power of 840 W.

## Abbreviations

The following abbreviations are used in this manuscript:

Ac	Acetyl
ASA	Alumina sulfuric acid
BTSA	Boric trisulfuric anhydride
bmim	1-Butyl-3-methylimidazolium
Bu	Butyl
CAN	Ceric ammonium nitrate
COD	1,5-Cyclooctadiene
Cp	Cyclopentadienyl
CSA	Cellulose sulfuric acid
Cy	Cyclohexyl
DBU	1,8-Diazabicyclo[5.4.0]undec-7-ene
DCE	1,2-Dichloroethane
DMA	Dimethylacetamide
DMF	*N*,*N*-Dimethylformamide
DMAP	4-(*N*,*N*-dimethylamino)pyridine
dppe	1,2-Bis(diphenylphosphino)ethane
dppb	1,4-Bis(diphenylphosphino)butane
dppf	1,1′-Bis(diphenylphosphino)ferrocene
Et	Ethyl
Me	Methyl
MSA	Molybdate sulfuric acid
MW	Microwave
Ph	Phenyl
Piv	Pivaloyl
PVSA	Polyvinyl sulfonic acid
Tf	Trifluoromethanesulfonyl
TFA	Trifluoroacetic acid
THF	Tetrahydrofuran
TMS	Trimethylsilyl
Ts	*p*-Toluenesulfonyl
US	Ultrasound
XSA	Xanthan sulfuric acid

## References

[1] Keri R.S., Budagumpi S., Pai R.K., Balakrishna R.G. (2014). Chromones as a privileged scaffold in drug discovery: A review. Eur. J. Med. Chem..

[2] Garazd M.M., Garazd Y.L., Khilya V.P. (2005). Neoflavones. 2. Methods for synthesizing and modifying 4-arylcoumarins. Chem. Nat. Comp..

[3] Wu J., Wang L., Fathi R., Yang Z. (2002). Palladium-catalyzed cross-coupling reactions of 4-tosylcoumarin and arylboronic acids: Synthesis of 4-arylcoumarin compounds. Tetrahedron Lett..

[4] Borges F., Roleira F., Milhazes N., Santana L., Uriarte E. (2005). Simple Coumarins and Analogues in Medicinal Chemistry: Occurrence, Synthesis and Biological Activity. Curr. Med. Chem..

[5] Medina F.G., Marrero J.G., Macías-Alonso M., González M.C., Córdova-Guerrero I., García S., Osegueda-Robles A.G.T. (2015). Coumarin heterocyclic derivatives: Chemical synthesis and biological activity. Nat. Prod. Rep..

[6] Gaudino E.C., Tagliapietra S., Martina K., Palmisano G., Cravotto G. (2016). Recent advances and perspectives in the synthesis of bioactive coumarins. RSC Adv..

[7] Stefanachi A., Leonetti F., Pisani L., Catto M., Carotti A. (2018). Coumarin: A Natural, Privileged and Versatile Scaffold for Bioactive Compounds. Molecules.

[8] Pechmann H.V. (1884). Neue Bildungsweise der Cumarine. Synthese des Daphnetins. Ber. Dtsch. Chem. Ges..

[9] Tyndall S., Wong K.F., VanAlstine-Parris M.A. (2015). Insight into the mechanism of the Pechmann condensation reaction using NMR. J. Org. Chem..

[10] Daru J., Stirling A. (2011). Mechanism of the Pechmann reaction: A theoretical study. J. Org. Chem..

[11] Pornsatitworakul S., Boekfa B., Maihom T., Treesukol P., Namuangruk S., Jarussophon S., Jarussophon N., Limtrakul J. (2017). The coumarin synthesis: A combined experimental and theoretical study. Monatsh. Chem..

[12] Zambare A.S., Kalam Khan F.A., Zambare S.P., Shinde S.D., Sangshetti J.N. (2016). Recent Advances in the Synthesis of Coumarin Derivatives via Pechmann Condensation. Curr. Org. Chem..

[13] Sugino T., Tanaka K. (2001). Solvent-free coumarin synthesis. Chem. Lett..

[14] Sharma D., Kumar S., Makrandi J. (2011). Modified Pechmann condensation using grinding technique under solvent-free condition at room temperature. Green Chem. Lett. Rev..

[15] Manhas M.S., Ganguly S.N., Mukherjee S., Jain A.K., Bose A.K. (2006). Microwave initiated reactions: Pechmann coumarin synthesis, Biginelli reaction, and acylation. Tetrahedron Lett..

[16] Singh P.R., Singh D.U., Samant S.D. (2004). Sulphamic acid-an efficient and cost-effective solid acid catalyst for the Pechmann reaction. Synlett.

[17] Moradi L., Rabiei K., Belali F. (2016). Meglumine sulfate catalyzed solvent-free one-pot synthesis of coumarins under microwave and thermal conditions. Synth. Commun..

[18] Karimi-Jaberi Z., Zarei L. (2013). Synthesis of coumarins and 2, 3-dihydroquinazolin-4 (1*H*)-ones. Acta Chim. Slov..

[19] Sunil Kumar B., Thirupathi Reddy Y., Narsimha Reddy P., Kumar P., Rajitha B. (2006). Selectfluor™: A simple and efficient catalyst for the synthesis of substituted coumarins under solvent-free conditions. J. Heterocycl. Chem..

[20] Sharma G., Reddy J.J., Lakshmi P.S., Krishna P.R. (2005). An efficient ZrCl_4_ catalyzed one-pot solvent free protocol for the synthesis of 4-substituted coumarins. Tetrahedron Lett..

[21] Kumar B.S., Kumar P., Srinivasulu N., Rajitha B., Reddy Y.T., Reddy P.N., Udupi R. (2006). Vanadium(III) chloride as an effective catalyst for the Pechmann reaction. Chem. Heterocycl. Compd..

[22] Naik M.A., Mishra B.G., Dubey A. (2007). A simple and efficient protocol for the synthesis of substituted comarins catalyzed by stannic(IV) chloride under solvent-free condition. React. Kinet. Catal. Lett..

[23] Wang Y., Xu F., Tian Y.-P., Li H.-L., Wang J.-J. (2009). CuX_2_ (X = Cl, Br) as catalysts for Pechmann reaction: Synthesis of 4-substituted coumarins. J. Chem. Res..

[24] Khodabakhshi S. (2012). Barium dichloride as a powerful and inexpensive catalyst for the Pechmann condensation without using solvent. Org. Chem. Int..

[25] Kumar S., Saini A., Sandhu J.S. (2007). LiBr-Mediated, solvent free von Pechmann reaction: Facile and efficient method for the synthesis of 2H-chromen-2-ones. Arkivoc.

[26] Patil S.B., Bhat R.P., Raje V.P., Samant S.D. (2006). Ultrasound-Assisted Pechmann Condensation of Phenols With β-Ketoesters to Form Coumarins, in the Presence of Bismuth(III) Chloride Catalyst. Synth. Commun..

[27] Madhav J.V., Kuarm B.S., Someshwar P., Rajitha B., Thirupathi Reddy Y.R., Crooks P.A. (2008). Dipyridine cobalt chloride: A novel catalyst for the synthesis of coumarins via Pechmann condensation. J. Chem. Res..

[28] Jung K., Park Y.-J., Ryu J.-S. (2008). Scandium(III) Triflate–Catalyzed Coumarin Synthesis. Synthetic Commun..

[29] Wang H. (2013). Magnesium bis(trifluoromethane)sulfonimide: An efficient catalyst for the synthesis of coumarins under solvent-free conditions. Monatsh. Chem..

[30] Alexander V.M., Bhat R.P., Samant S.D. (2005). Bismuth(III) nitrate pentahydrate—A mild and inexpensive reagent for synthesis of coumarins under mild conditions. Tetrahedron Lett..

[31] Reddy Y.T., Sonar V.N., Crooks P.A., Dasari P.K., Reddy P.N., Rajitha B. (2008). Ceric ammonium nitrate (CAN): An efficient catalyst for the coumarin synthesis via Pechmann condensation using conventional heating and microwave irradiation. Synth. Commun..

[32] Karami B., Kiani M. (2014). Synthesis of the Coumarins via Pechmann Method in the Presence of Environmentally Friendly Y(NO_3_) _3_× 6H_2_O. J. Chin. Chem. Soc..

[33] Soleimani E., Khodaei M.M., Batooie N., Samadi S. (2012). Tetrakis(acetonitrile)copper(I) hexafluorophosphate catalyzed coumarin synthesis via pechmann condensation under solvent-free condition. J. Heterocycl. Chem..

[34] Jafari F., Khodabakhshi S. (2014). MnSO_4_. H_2_O: A highly efficient and inexpensive catalyst for the synthesis of benzo-2-pyrones and benzopyrazines. Bulg. Chem. Commun..

[35] Moradi L., Belali F. (2015). Solvent-free one-pot synthesis of coumarins using molybdate sulfuric acid as highly efficient catalyst. J. Iran. Chem. Soc..

[36] Goswami P. (2009). Dually activated organo-and nano-cocatalyzed synthesis of coumarin derivatives. Synthetic Commun..

[37] Naik M.A., Mishra B.G., Dubey A. (2008). Combustion synthesized WO_3_–ZrO_2_ nanocomposites as catalyst for the solvent-free synthesis of coumarins. Colloids Surf. A..

[38] Wu L.-Q., Yan F.-L., Yang C.-G., Yang L.-M. (2010). Silica Chloride Catalyzed Solvent-Free Synthesis of Coumarins. Chin. J. Org. Chem..

[39] Sharma D., Kumar S. (2013). A facile synthesis of 2H-chromen-2-ones via Pechmann condensation under solvent free conditions using grinding technique. Green Process. Synth..

[40] Rad-Moghadam K., Montazeri N. (2009). Coumarin synthesis via pechmann condensation on silica-supported sulfuric acid under microwave irradiation. Asian J. Chem..

[41] Karami B., Kiani M. (2011). ZrOCl_2_. 8H_2_O/SiO_2_: An efficient and recyclable catalyst for the preparation of coumarin derivatives by Pechmann condensation reaction. Catal. Commun..

[42] Parhami A., Khalafi-Nezhad A., Haghighi S.M., Bargebid R., Zare A., Moosavi-Zare A.R., Nikrooz M. (2012). Silica supported boric tri-sulfuric anhydride as a novel and efficient catalyst for solvent-free synthesis of coumarins via Pechmann condensation. Arkivoc.

[43] Sun R., Gao Y., Ma Y., Yang G., Li Y. (2017). SnCl_4_ grafted on silica gel: An efficient catalyst for solvent-free synthesis of coumarins via the Pechmann condensation. J. Iran. Chem. Soc..

[44] Amoozadeh A., Ahmadzadeh M., Kolvari E. (2012). Easy access to coumarin derivatives using alumina sulfuric acid as an efficient and reusable catalyst under solvent-free conditions. J. Chem..

[45] Kuarm B.S., Crooks P.A., Rajitha B. (2012). Polyvinylsulfonic acid: An Efficient and Recyclable Bronsted Acid Catalyst for Pechmann Condensation. Int. J. Res. Pharm. Biomed. Sci..

[46] Kuarm B.S., Madhav J.V., Rajitha B. (2012). Xanthan sulfuric acid: An efficient and recyclable solid acid catalyst for Pechmann condensation. Synthetic Commun..

[47] Kuarm B.S., Madhav J.V., Laxmi S.V., Rajitha B., Reddy Y.T., Reddy P.N., Crooks P.A. (2010). Expeditious pechmann condensation by using biodegradable cellulose sulfuric acid as a solid acid catalyst. Synth. Commun..

[48] Mobaraki A., Yasham S., Movassagh B. (2015). Environmentally Sustainable Magnetic Solid Sulfonic Acid: An Efficient and Reusable Catalyst for the Pechmann Reaction. Synlett.

[49] Abbasi Z., Rezayati S., Bagheri M., Hajinasiri R. (2017). Preparation of a novel, efficient, and recyclable magnetic catalyst, γ-Fe_2_O_3_@HAp-Ag nanoparticles, and a solvent-and halogen-free protocol for the synthesis of coumarin derivatives. Chin. Chem. Lett..

[50] Dabiri M., Baghbanzadeh M., Kiani S., Vakilzadeh Y. (2007). Alum (KAl(SO_4_)_2_·12H_2_O) Catalyzed One-Pot Synthesis of Coumarins under Solvent-Free Conditions. Monatsh. Chem..

[51] Azizian J., Mohammadi A.A., Bidar I., Mirzaei P. (2008). KAl(SO_4_)_2_·12H_2_O (alum) a reusable catalyst for the synthesis of some 4-substituted coumarins *via Pechmann* reaction under solvent-free conditions. Monatsh. Chem..

[52] Kalyanam N., Nagarajan A., Majeed M. (2004). A Single-Step Assembly of Coumarin Ring Skeleton from Oxygenated Phenols and Acetylenic Esters by Catalytic Indium Chloride in the Absence of Solvent. Synth. Commun..

[53] Leao R.A., de Moraes P.D.F., Pedro M.C., Costa P.R. (2011). Synthesis of coumarins and neoflavones through zinc chloride catalyzed hydroarylation of acetylenic esters with phenols. Synthesis.

[54] Escobar A.M., Ruiz D.M., Autino J.C., Romanelli G.P. (2015). Single-step synthesis of 4-phenyl and 3,4-dihydro-4-phenyl coumarins using a recyclable Preyssler heteropolyacid catalyst under solvent-free reaction conditions. Res. Chem. Intermediat..

[55] Li R., Wang S.R., Lu W. (2007). FeCl_3_-catalyzed alkenylation of simple arenes with aryl-substituted alkynes. Org. Lett..

[56] Kutubi M.S., Hashimoto T., Kitamura T. (2011). Improved synthesis of coumarins by iron(III)-catalyzed cascade reaction of propiolic acids and phenols. Synthesis.

[57] Fiorito S., Epifano F., Taddeo V.A., Genovese S. (2016). Ytterbium triflate promoted coupling of phenols and propiolic acids: Synthesis of coumarins. Tetrahedron Lett..

[58] Bennardi D.O., Ruiz D.M., Romanelli G.P., Baronetti G.T., Thomas H.J., Autino J.C. (2008). Efficient microwave solvent-free synthesis of flavones, chromones, coumarins and dihydrocoumarins. Lett. Org. Chem..

[59] Song C.E., Jung D.U., Choung S.Y., Roh E.J., Lee S.G. (2004). Dramatic enhancement of catalytic activity in an ionic liquid: Highly practical friedel–crafts alkenylation of arenes with alkynes catalyzed by metal triflates. Angew. Chem. Int. Ed..

[60] Yoon M.Y., Kim J.H., Choi D.S., Shin U.S., Lee J.Y., Song C.E. (2007). Metal Triflate-Catalyzed Regio-and Stereoselective Friedel–Crafts Alkenylation of Arenes with Alkynes in an Ionic Liquid: Scope and Mechanism. Adv. Synth. Catal..

[61] Trost B.M., Toste F.D. (1996). A new palladium-catalyzed addition: A mild method for the synthesis of coumarins. J. Am. Chem. Soc..

[62] Trost B.M., Toste F.D., Greenman K. (2003). Atom Economy. Palladium-Catalyzed Formation of Coumarins by Addition of Phenols and Alkynoates via a Net C−H Insertion. J. Am. Chem. Soc..

[63] Jia C., Piao D., Kitamura T., Fujiwara Y. (2000). New Method for Preparation of Coumarins and Quinolinones via Pd-Catalyzed Intramolecular Hydroarylation of C−C Triple Bonds. J. Org. Chem..

[64] Jia C., Piao D., Oyamada J., Lu W., Kitamura T., Fujiwara Y. (2000). Efficient activation of aromatic C–H bonds for addition to C–C multiple bonds. Science.

[65] Oyamada J., Jia C., Fujiwara Y., Kitamura T. (2002). Direct synthesis of coumarins by Pd(II)-catalyzed reaction of alkoxyphenols and alkynoates. Chem. Lett..

[66] Kotani M., Yamamoto K., Oyamada J., Fujiwara Y., Kitamura T. (2004). A convenient synthesis of coumarins by palladium(II)-catalyzed reaction of phenols with propiolic acids. Synthesis.

[67] Oyamada J., Kitamura T. (2006). Synthesis of coumarins by Pt-catalyzed hydroarylation of propiolic acids with phenols. Tetrahedron.

[68] Shi Z., He C. (2004). Efficient Functionalization of Aromatic C−H Bonds Catalyzed by Gold(III) under Mild and Solvent-Free Conditions. J. Org. Chem..

[69] Gadakh S.K., Dey S., Sudalai A. (2015). Rh-Catalyzed Synthesis of Coumarin Derivatives from Phenolic Acetates and Acrylates via C–H Bond Activation. J. Org. Chem..

[70] Wu X.-F., Neumann H., Beller M. (2012). Synthesis of heterocycles via palladium-catalyzed carbonylations. Chem. Rev..

[71] Zhang Z., Ju T., Miao M., Han J.-L., Zhang Y.-H., Zhu X.-Y., Ye J.-H., Yu D.-G., Zhi Y.-G. (2017). Transition-Metal-Free Lactonization of sp2 C–H Bonds with CO_2_. Org. Lett..

[72] Ferguson J., Zeng F., Alper H. (2012). Synthesis of coumarins via Pd-catalyzed oxidative cyclocarbonylation of 2-vinylphenols. Org. Lett..

[73] Liu X.-G., Zhang S.-S., Jiang C.-Y., Wu J.-Q., Li Q., Wang H. (2015). Cp*Co(III)-Catalyzed Annulations of 2-Alkenylphenols with CO: Mild Access to Coumarin Derivatives. Org. Lett..

[74] Sakakura T., Choi J.-C., Yasuda H. (2007). Transformation of carbon dioxide. Chem. Rev..

[75] Tsuji Y., Fujihara T. (2012). Carbon dioxide as a carbon source in organic transformation: Carbon–carbon bond forming reactions by transition-metal catalysts. Chem. Commun..

[76] Aresta M., Dibenedetto A., Angelini A. (2013). Catalysis for the valorization of exhaust carbon: From CO_2_ to chemicals, materials, and fuels. Technological use of CO_2_. Chem. Rev..

[77] Sasano K., Takaya J., Iwasawa N. (2013). Palladium(II)-catalyzed direct carboxylation of alkenyl C–H bonds with CO_2_. J. Am. Chem. Soc..

[78] Bissel P., Nazih A., Sablong R., Lepoittevin J.-P. (1999). Stereoselective synthesis of (R)-and (S)-4-methoxydalbergione via asymmetric catalytic hydrogenation. Org. Lett..

[79] Harikrishna K., Rakshit A., Aidhen I.S. (2013). Study of the chemoselectivity of Grignard reagent addition to substrates containing both nitrile and Weinreb amide functionalities. Eur. J. Org. Chem..

[80] Gallagher B.D., Taft B.R., Lipshutz B.H. (2009). Asymmetric conjugate reductions of coumarins. A new route to tolterodine and related coumarin derivatives. Org. Lett..

[81] Wang D., Cui S. (2015). Rh(III)-catalyzed aldehyde C–H bond functionalization of salicylaldehydes with arylboronic acids. Tetrahedron.

[82] Upadhyay P.K., Kumar P. (2009). A novel synthesis of coumarins employing triphenyl (α-carboxymethylene) phosphorane imidazolide as a C-2 synthon. Tetrahedron Lett..

[83] Taylor R.T., Cassell R.A. (1982). Trimethylsilylketene: Synthesis of coumarins via cyclization-elimination. Synthesis.

[84] Sethna S.M., Shah N.M. (1945). The Chemistry of Coumarins. Chem. Rev..

[85] Kawase Y., Yamaguchi S., Aoyama K., Matsuda M. (1978). The Fries Rearrangement of bz-Benzoyloxybenzofuran Derivatives and the Synthesis of the Furo Derivatives of 4-Phenyl-2H-chromen-2-one. B. Chem. Soc. Jpn..

[86] Gaspar A., Matos M.J.O., Garrido J., Uriarte E., Borges F. (2014). Chromone: A valid scaffold in medicinal chemistry. Chem. Rev..

[87] Hwang I.-T., Lee S.-A., Hwang J.-S., Lee K.-I. (2011). A facile synthesis of highly functionalized 4-arylcoumarins via kostanecki reactions mediated by DBU. Molecules.

[88] Crecente-Campo J., Pilar Vázquez-Tato M., Seijas J.A. (2010). Microwave-Promoted, One-Pot, Solvent-Free Synthesis of 4-Arylcoumarins from 2-Hydroxybenzophenones. Eur. J. Org. Chem..

[89] Sridhar Reddy M., Thirupathi N., Babu M.H. (2012). Synthesis of Substituted Coumarins and 2-Quinolinones by Cycloisomerisation of (Hydroxy/aminophenyl) propargyl Alcohols. Eur. J. Org. Chem..

[90] Li J., Chen H., Zhang-Negrerie D., Du Y., Zhao K. (2013). Synthesis of coumarins via PIDA/I_2_-mediated oxidative cyclization of substituted phenylacrylic acids. RSC Adv..

[91] Wattanasin S. (1988). Novel Route to 4-Aryl Coumarins. Synth. Commun..

[92] Schio L., Chatreaux F., Klich M. (2000). Tosylates in palladium-catalysed coupling reactions. Application to the synthesis of arylcoumarin inhibitors of gyrase B. Tetrahedron Lett..

[93] Ciattini P.G., Morera E., Ortar G. (1995). 4-Stannylcoumarins as Useful Reagents in a New Approach to Neoflavonoids. Synth. Commun..

[94] Lee D.-H., Qian Y., Park J.-H., Lee J.-S., Shim S.-E., Jin M.-J. (2013). A Highly Active and General Catalyst for the Stille Coupling Reaction of Unreactive Aryl, Heteroaryl, and Vinyl Chlorides under Mild Conditions. Adv. Synth. Catal..

[95] Boland G.M., Donnelly D.M.X., Finet J.P., Rea M.D. (1996). Synthesis of neoflavones by Suzuki arylation of 4-substituted coumarins. J. Chem. Soc. Perkin Trans. 1.

[96] Chen L., Hu T.S., Yao Z.J. (2008). Development of New Pyrrolocoumarin Derivatives with Satisfactory Fluorescent Properties and Notably Large Stokes Shifts. Eur. J. Org. Chem..

[97] Wu J., Zhang L., Xia H.-G. (2006). Palladium-catalyzed Suzuki-Miyaura coupling of potassium aryl trifluoroborates with 4-tosyloxycoumarins or 4-tosyloxyquinolin-2(1*H*)-one. Tetrahedron Lett..

[98] Zhang L.A., Meng T.H., Fan R.H., Wu H. (2007). General and efficient route for the synthesis of 3,4-disubstituted coumarins via Pd-catalyzed site-selective cross-coupling reactions. J. Org. Chem..

[99] Akrawi O.A., Nagy G.Z., Patonay T., Villinger A., Langer P. (2012). Chemoselective Suzuki–Miyaura reactions of 4-trifluoromethylsulfonyloxy-6-bromocoumarin. Tetrahedron Lett..

[100] Khaddour Z., Akrawi O.A., Suleiman A.S., Patonay T., Villinger A., Langer P. (2014). Regioselective Suzuki-Miyaura cross-coupling reactions of the bis(triflate) of 4,7-dihydroxycoumarin. Tetrahedron Lett..

[101] Rajale T., Sharma S., Stroud D.A., Unruh D.K., Miaou E., Lai K., Birney D.M. (2014). An efficient synthesis of 4-substituted coumarin derivatives via a palladium-catalyzed Suzuki cross-coupling reaction. Tetrahedron Lett..

[102] Wu J., Liao Y., Yang Z. (2001). Synthesis of 4-substituted coumarins via the palladium-catalyzed cross-couplings of 4-tosylcoumarins with terminal acetylenes and organozinc reagents. J. Org. Chem..

[103] Rieke R.D., Kim S.H. (2011). A novel organozinc reagent 4-coumarinylzinc bromide; preparation and application in the synthesis of 4-substituted coumarin derivatives. Tetrahedron Lett..

[104] Rao M.L.N., Venkatesh V., Jadhav D.N. (2010). Palladium-Catalyzed Synthesis of 4-Arylcoumarins Using Triarylbismuth Compounds as Atom-Efficient Multicoupling Organometallic Nucleophiles. Eur. J. Org. Chem..

[105] Rao M.L.N., Kumar A. (2014). Pd-catalyzed chemo-selective mono-arylations and bis-arylations of functionalized 4-chlorocoumarins with triarylbismuths as threefold arylating reagents. Tetrahedron.

[106] Shah P., Santana M.D., Garcia J., Serrano J.L., Naik M., Pednekar S., Kapdi A.R. (2013). [Pd(PPh_3_)_2_(saccharinate)_2_]-general catalyst for Suzuki-Miyaura, Negishi cross-coupling and C–H bond functionalization of coumaryl and pyrone substrates. Tetrahedron.

[107] Wu J., Yang Z. (2001). Nickel-catalyzed cross-couplings of 4-diethylphosphonooxycoumarins with organozinc reagents: An efficient new methodology for the synthesis of 4-substituted coumarins. J. Org. Chem..

[108] Lei J.G., Xu M.H., Lin G.Q. (2004). Nickel-catalyzed cross-coupling reactions of 4-mesylcoumarins with aryl halides: Facile synthesis of 4-substituted coumarins. Synlett..

[109] Tang Z.Y., Hu Q.S. (2004). Room temperature nickel(0)-catalyzed Suzuki-Miyaura cross-couplings of activated alkenyl tosylates: Efficient synthesis of 4-substituted coumarins and 4-substituted 2(5*H*)-furanones. Adv. Synth. Catal..

[110] Xing C.H., Lee J.R., Tang Z.Y., Zheng J.R., Hu Q.S. (2011). Room Temperature Nickel(II) Complexes [(4-MeOC_6_H_4_)Ni(PCy_3_)_2_OTs and Ni(PCy_3_)_2_X_2_]-Catalyzed Cross-Coupling Reactions of Aryl/Alkenyl Sulfonates with Arylboronic Acids. Adv. Synth. Catal..

[111] Xu L., Li B.J., Wu Z.H., Lu X.Y., Guan B.T., Wang B.Q., Zhao K.Q., Shi Z.J. (2010). Nickel-Catalyzed Efficient and Practical Suzuki-Miyaura Coupling of Alkenyl and Aryl Carbamates with Aryl Boroxines. Org. Lett..

[112] Wu J., Zhang L., Gao K. (2006). RhCl(PPh_3_)_3_/DPPF: A useful and efficient catalyst for cross-coupling reactions of activated alkenyl tosylates with arylboronic acids. Eur. J. Org. Chem..

[113] Wu J., Zhang L., Luo Y. (2006). Rh(I)-catalyzed cross-coupling reactions of alkenyl tosylates with potassium aryltrifluoroborates. Tetrahedron Lett..

[114] Khoobi M., Alipour M., Zarei S., Jafarpour F., Shafiee A. (2012). A facile route to flavone and neoflavone backbones via a regioselective palladium catalyzed oxidative Heck reaction. Chem. Commun..

[115] Li Y.M., Qi Z.S., Wang H.F., Fu X.M., Duan C.Y. (2012). Palladium-Catalyzed Oxidative Heck Coupling Reaction for Direct Synthesis of 4-Arylcoumarins Using Coumarins and Arylboronic Acids. J. Org. Chem..

[116] Min M., Hong S. (2012). Regioselective palladium-catalyzed direct cross-coupling of coumarins with simple arenes. Chem. Commun..

[117] Kang D., Ahn K., Hong S. (2018). Site-Selective C−H Bond Functionalization of Chromones and Coumarins. Asian J. Org. Chem..

[118] Khoobi M., Molaverdi F., Alipour M., Jafarpour F., Foroumadi A., Shafiee A. (2013). Palladium-catalyzed domino protodecarboxylation/oxidative Heck reaction: Regioselective arylation of coumarin-3-carboxylic acids. Tetrahedron.

[119] Battistuzzi G., Cacchi S., Salve I.D., Fabrizi G., Parisi L.M. (2005). Synthesis of Coumarins in a Molten n-Bu_4_NOAc/n-Bu_4_NBr Mixture through a Domino Heck Reaction/Cyclization Process. Adv. Synth. Catal..

[120] Barancelli D.A., Salles A.G., Taylor J.G., Correia C.R. (2012). Coumarins from free ortho-hydroxy cinnamates by Heck-Matsuda arylations: A scalable total synthesis of (R)-tolterodine. Org. Lett..

[121] Yang Y., Han J., Wu X., Xu S., Wang L. (2015). Synthesis of 4-arylcoumarins via palladium-catalyzed arylation/cyclization of ortho-hydroxylcinnamates with diaryliodonium salts. Tetrahedron Lett..

[122] Perez J.M., Cano R., McGlacken G.P., Ramon D.J. (2016). Palladium(II) oxide impregnated on magnetite as a catalyst for the synthesis of 4-arylcoumarins via a Heck-arylation/cyclization process. RSC Adv..

[123] Yamamoto Y., Kirai N. (2008). Synthesis of 4-arylcoumarins via Cu-catalyzed hydroarylation with arylboronic acids. Org. Lett..

